# Sarcopenia-related traits and risk of falls in older adults: results from meta-analysis of cohort studies and Mendelian randomization analyses

**DOI:** 10.1007/s40520-025-02997-7

**Published:** 2025-03-26

**Authors:** Haohan Yang, Yu Jiang, Dingfa Liang, Chang Yang, Kaihua Qin, Yong Xie, Licheng Zhang, Peifu Tang, Xiang Cui, Houchen Lyu

**Affiliations:** 1 Medical School of Chinese PLA, Beijing, 100853 China; 2 Department of Orthopedics, Chinese PLA General Hospital, No. 28, Fuxing Road, Beijing, 100853 China; 3 National Clinical Research Center for Orthopedics, Sports Medicine & Rehabilitation, Beijing, 100853 China

**Keywords:** Sarcopenia-related traits, Falls, Meta-analysis, Mendelian randomization study

## Abstract

**Background:**

Observational studies examining sarcopenia-related traits and fall risk remain controversial. Herein, we conducted meta-analyses of cohort studies triangulated with Mendelian randomization (MR) analyses to examine the potential causality between sarcopenia-related traits and risk of falls in older adults.

**Methods:**

Literature search across PubMed, Embase, and Cochrane Library was performed from inception to February 2023 to identify cohort studies examining sarcopenia-related traits (including hand strength, appendicular lean mass, and walking speed) and falls. We assessed the association between these traits and fall risk using random-effects models to calculate pooled odds ratios (OR) and 95% confidence intervals (CIs). MR analyses were conducted using summary statistics derived from the UK Biobank consortium for sarcopenia-related traits and FinnGen consortium for falls. The inverse-variance weighted method was used as primary analysis.

**Results:**

Our meta-analysis included 34 cohort studies. The combined analysis of sarcopenia-related traits revealed a 33% reduced fall risk with each unit increase in walking speed (OR 0.67, 95% CI 0.54–0.84) and a 2% decrease with each unit increase in hand strength (OR 0.98, 95% CI 0.97–0.99). However, appendicular lean mass had no significant effect on falls. In the MR analyses, only walking speed was causally associated with falls (OR 0.64, 95% CI 0.48–0.84). Hand strength and appendicular lean mass showed no statistically significant causal effect on falls.

**Conclusion:**

Evidence from meta-analysis and MR suggests a strong association between walking speed and fall risk in older adults. However, the relationship between hand strength, appendicular lean mass, and falls has not yet been established.

**Supplementary Information:**

The online version contains supplementary material available at 10.1007/s40520-025-02997-7.

## Introduction

Falls are common and serious concern among older adults, with one in three adults aged ≥ 65 years falling at least once a year [1]. In the United States, falls are the leading cause of injury-related death among older adults, accounting for 46.2% [2]. They are also linked to function decline, loss of independence, and increased mortality rates [3], leading to a substantial economic burden. The estimated medical costs associated with falls in the healthcare system are around $50 billion each year [4]. Identifying modifiable risk factors is crucial for reducing the incidence of falls among older adults.

Sarcopenia, characterized by decreased muscle mass and function, is considered an important modifiable risk factor for falls, with a global prevalence ranging from 3.3% to 36% [5–8]. Key indicators of sarcopenia-related traits include hand strength, appendicular lean mass, and walking speed [9]. However, the relationship between these traits and falls remains inconsistent. A cross-sectional study including 3901 older participants found that people with low hand strength had an increased risk of falls (OR 1.23, 95% CI 1.02–1.49) [10], and a prospective study involving 4987 older adults reported similar findings (OR 1.18, 95% CI 1.00–1.40) [11]. However, a prospective analysis including 15,103 participants failed to find an association between low hand strength and fall risk (OR 0.99, 95% CI 0.74–1.32) [12]. These discrepancies may stem from differences in sample sizes across studies, as well as the inherent limitations of observational studies, such as unmeasured confounders.

To overcome these challenges, the combination of meta-analysis and Mendelian randomization (MR) offers significant advantages. A meta-analysis of all available cohort studies would enhance statistical power by effectively increasing the sample size and resolving inconsistencies in findings by synthesizing data from multiple sources. Meanwhile, MR uses genetic variants as instrumental variables (IVs) to estimate the causal effect of exposure, providing a unique advantage of mitigating the effect of unmeasured confounding and reverse causality [13–15]. This approach has been extensively applied to investigate the causal relationships in various contexts, such as between kidney function and dementia, glucose level and ischemic stroke, and psoriasis and cardiovascular disease [16–18]. Applying this combined design to the study sarcopenia-related traits and falls would enable us to address the two primary challenges of observational studies (i.e., inadequate power and unmeasured confounder) for this clinical important question, thereby providing more reliable evidence to inform clinical decision-making.

In this study, we performed a meta-analysis of cohort studies triangulated with MR to give a comprehensive evaluation of the potential causal association between sarcopenia-related traits and risk of falls in older adults.

## Methods

### Meta-analysis

#### Literature search

This study followed the Preferred Reporting Items for Systematic Reviews and Meta-Analyses (PRISMA) guideline and the study protocol was registered at PROSPERO (CRD42024517898) [19]. Two authors (HY and CY) independently conducted a literature search of the PubMed, Embase, and Cochrane Library databases from their inception through February 2023. Search strategies were developed using text words and medical subject headings (MeSH) related to the outcomes of interest (falls) and the exposures of interest (hand strength, appendicular lean mass, and walking speed), together with the term “Sarcopenia”. The full search strategies used in Pubmed, Embase, and Cochrane Library can be found in the supplementary materials (Supplementary Table 1–4).

### Study selection

Studies included in the meta-analysis were required to meet the following inclusion criteria regarding the study design, study population, exposure, and outcome of interest. These criteria were: (i) cohort studies; (ii) a mean or median age of ≥ 65 years; (iii) research into the relationship between sarcopenia-related traits (hand strength, appendicular lean mass, and walking speed) and falls, with results presented as odds ratios/risk ratios/hazard ratios and 95% confidence intervals; (iv) published in English. Studies published as conference abstracts, reviews, editorials, letters to the editor, and case reports were excluded. In cases where studies included overlapping cohorts, the one with the largest number of participants was chosen. Two review authors (HY and CY) independently reviewed the titles and abstracts for eligibility, with disagreements resolved by a third reviewer (YJ). Studies that did not meet the eligibility criteria were excluded.

### Data extraction and quality assessment

After removing duplicated and irrelevant articles, the full text of the remaining studies was evaluated and the following information was extracted independently by two authors (HY and CY): author, publication year, total number of participants included in the study, mean/median age of participants, fall follow-up duration, percentage of females, population, continent, incidence of falls, types of exposure variables, cut-off points of exposure, and risk of falls. Disagreements were resolved through discussion.

The risk of bias was assessed with the Newcastle Ottawa Scale (NOS) by two authors (HY and CY) independently [20]. The scale assigns points to specific categories: (i) selection of the study population, (ii) comparability, and (iii) description of the outcome. Each study could receive a maximum of one point per item within the selection and outcome categories and up to two points for the comparability category. The total scale score for cohort studies ranges from 0 to 9, with studies scoring ≥ 7 points considered high quality [5].

### Data synthesis and analysis

We standardized effect estimates by converting RR or HR values into ORs according to previous literature (Supplementary Information) [21]. Changes in fall risk per standard deviation (SD) increase in sarcopenia-related traits were recalculated as per unit increase, assuming a linear exposure–response relationship, and the following formula was used to recalculate the OR and 95% CI ${\text{OR}}_{\text{Standardized }}= \text{ exp } \left[\frac{LN\left({OR}_{original}\right)}{original increment} \times standardized increment\right]$ [22].

Effect sizes were visualized using forest plots. Heterogeneity was assessed using the chi-square test for Cochrane’s Q statistic and calculating I^2^, with significant heterogeneity defined as I^2^ > 50%. Additionally, Tau^2^ was calculated to estimate the absolute variability in effect sizes across studies. The random-effects model was used due to the anticipated presence of significant heterogeneity [23]. Sensitivity analyses evaluated whether the results could have been affected markedly by a single study, and subgroup analysis was conducted based on the population, continent, follow-up time, study quality, sex, and walking test distance. We assessed for possible publication bias using funnel plot, Egger regression, and the trim and fill method [24–26]. Meta-analysis analyses were performed using R (version 4.1.0) with “meta” (v.6.5-0) and “metafor” (v.4.4-0) packages. *P* values < 0.05 were considered statistically significant (two-tailed).

## Mendelian randomization

**Fig. 1:**
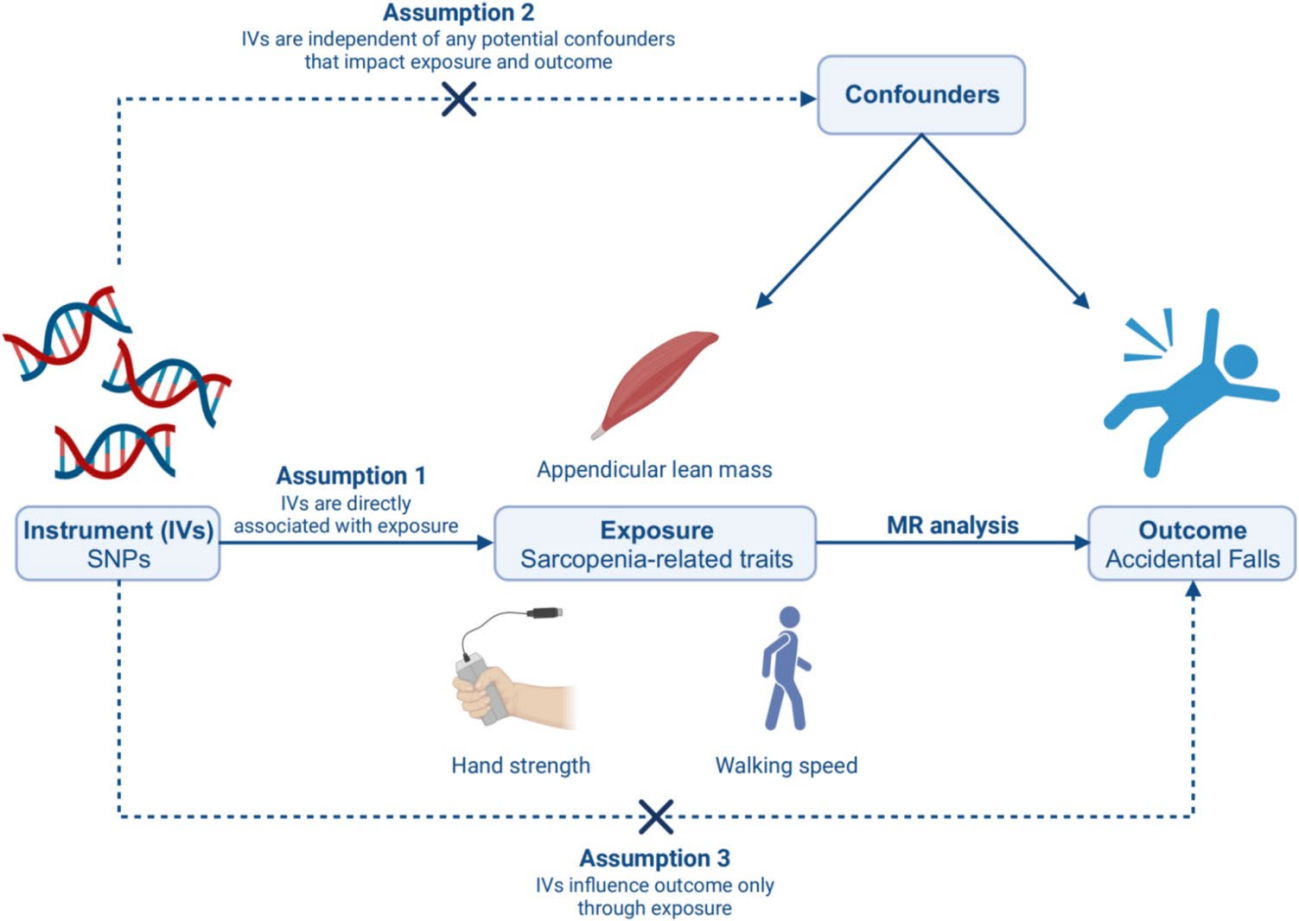
Overview of the MR design. *MR* Mendelian randomization, *IVs* instrumental variables, *SNPs* single nucleotide polymorphisms

We conducted a two-sample MR analysis using summary-level statistics from genome-wide association studies (GWAS) to examine the potential causal relationship between sarcopenia-related traits and falls. The MR method is based on three main assumptions, as summarized in Fig. 1.

Our study followed the STROBE-MR reporting guidelines [27]. No additional ethical approval was required for our study because all analyses were only based on publicly available summary statistics.

### Data sources

We obtained summary-level GWAS data for sarcopenia-related traits from the UK Biobank consortium and data for falls from the FinnGen consortium. Detailed sources are listed in Supplementary Table 8. The summary-level GWAS for hand strength, appendicular lean mass, and walking speed involved 461,026, 450,243, and 459,915 participants, respectively. The summary-level GWAS for falls involved 377,277 participants of European ancestry from the FinnGen study.

### Selection of instrumental variables

To ensure the validity of IVs, we rigorously conducted a series of quality control procedures. The SNP loci derived from the GWAS summary data on hand strength, appendicular lean mass, and walking speed met the criterion of genome-wide significance (*P* < 5 × 10^–8^). We then assessed the linkage disequilibrium (LD) using data from the 1000 Genomes Project European sample (R^2^ = 0.001, window size = 10,000 kb) for clumping and removed SNPs with minor allele frequency (MAF) < 0.01. Moreover, based on directed acyclic graph (DAG) and prior knowledge, we examined each sarcopenia-related trait SNP for potential pleiotropic effects with common confounders (including alcohol consumption, self-health status, diabetes, eye diseases, joint diseases, neurological disorders, and use of antihyperglycemic drugs) (Supplementary Fig. 8). Following these steps, SNPs were selected from the outcome GWAS dataset, and missing SNPs were excluded from the analysis. Ambiguous palindromic SNPs were harmonized to prevent bias in causal estimation.

Finally, we calculated the F-statistic for SNPs, excluding those with an F statistic < 10 to mitigate the impact of the weak instrumental bias [28].

### Statistical analysis

For the primary MR analysis, we utilized the inverse variance-weighted (IVW) method. Sensitivity analyses, including MR-PRESSO, MR-Egger, weighted-median, and weighted-mode methods, were conducted to test the robustness of the findings. The IVW method combines the Wald ratio estimate for each SNP into a causal estimate for each risk factor, providing more robust causal estimates in the absence of pleiotropy [29]. MR-PRESSO identifies and addresses horizontal pleiotropy by removing significant outliers [30]. MR Egger relaxes the assumption of no horizontal pleiotropy and allows for a nonzero intercept, providing robust causal effects even in the presence of violated no horizontal assumptions [31]. The weighted-median provides a consistent assessment of causality when up to 50% weights are from invalid instruments [32]. The weighted-mode method uses the mode of the IVW empirical density function as the effect estimate and is robust to horizontal pleiotropy [33]. The MR-Egger intercept tests were used to assess the potential horizontal pleiotropy [31]. Cochran’s Q test was used to quantify the heterogeneity among the causal estimates of different genetic variants [34]. Additionally, we used the MR Steiger test to infer the causal direction between sarcopenia-related traits and falls [35].

The two-sample MR was analyzed using R (version 4.1.0) with the “TwoSampleMR” (v.0.5.6), “MendelianRandomization” (v.0.9.0) and “MRPRESSO” (v1.0) packages. *P* values < 0.05 were considered statistically significant (two-tailed).

## Results

### Meta-analysis

#### Literature search

**Fig. 2:**
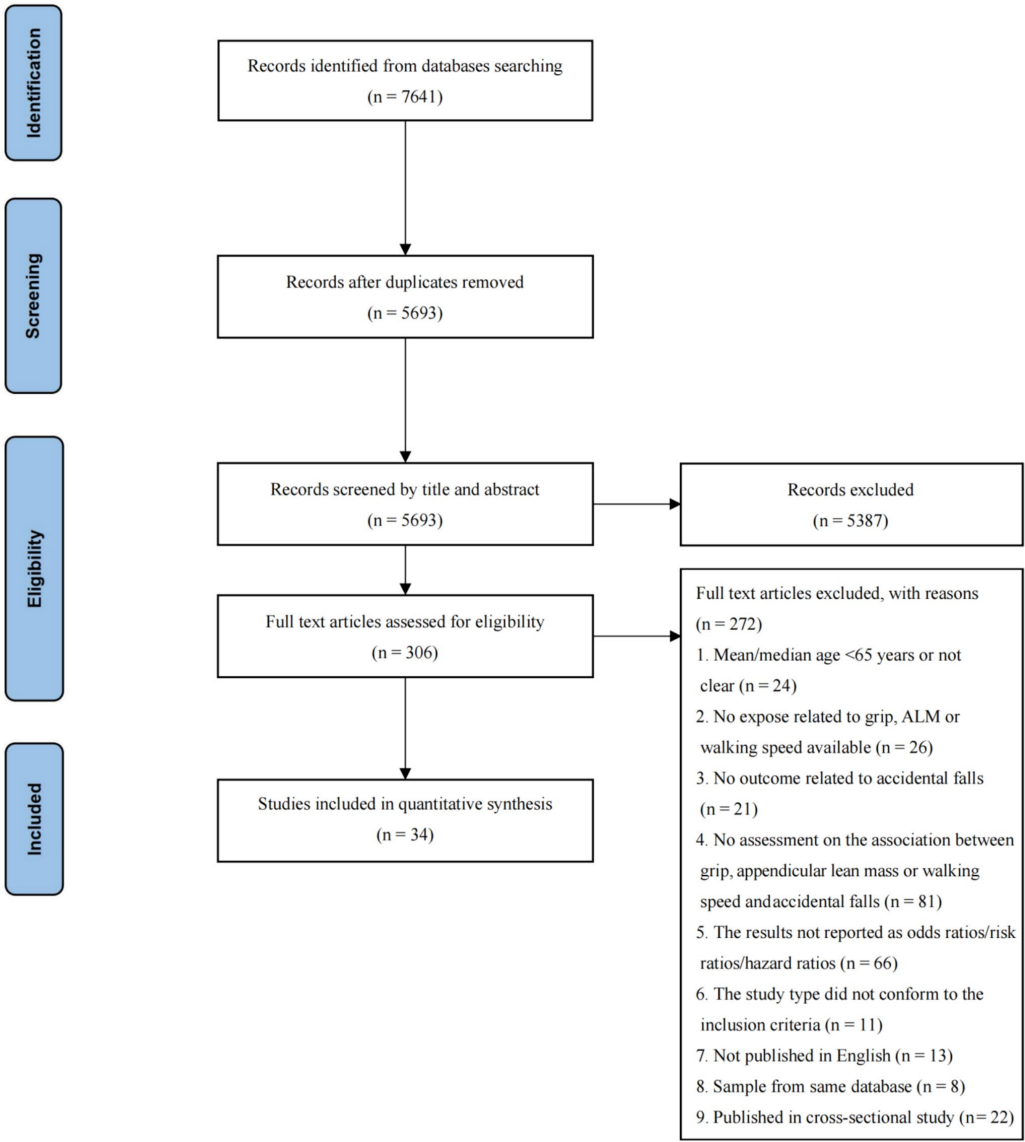
The flow diagram of study identification and selection for meta-analysis

We identified 5693 records after removing duplicates. Following a series of screenings, 34 studies were eligible for data extraction and quantitative analysis [11, 12, 36–67] (Fig. 2).

#### Study characteristics

Table 1 and Supplementary Table 5 summarize the characteristics of the included studies. A total of 57,449 participants (53.9% females) were included, with mean or median ages ranging from 65.0 to 88.0 years. Sample sizes ranged from 24 to 15,103 participants, and follow-up periods ranged from 3 to 168 months. The incidence of falls varied from 6.7% to 74.3%. Study populations included community-dwelling participants (n = 26) and hospitalized/outpatients/disease patients (n = 8). Most studies were conducted in Europe (n = 19), followed by Asia (n = 8), North America (n = 5), Oceania (n = 1) and multiple regions (n = 1). Moreover, we present in Supplementary Table 6 the covariates adjusted for each of the studies included in our analysis.

**Table 1:** Study characteristics of sarcopenia-related traits as continuous variables and fall outcomes

Author	Year	N	Mean/MEDIAN age ± SD (years)	Follow-up time (months)	Female, n (%)	Population	Continent	Falls incidence, n (%)
Hand strength								
Ancum	2018	297	79.3 ± 6.1	3	156 (52.5%)	Hospital patients/outpatients	Europe	58 (19.5%)
Arvandi	2018	808	75.0 ± 6.0	12	399 (49.4%)	Community	Europe	145 (17.9%)
Dowling	2023	4239	69.4 ± 6.6	24	2279 (53.8%)	Community	Europe	1049 (24.7%)
Inose	2021	135	68.6 ± 10.2	12	51 (37.8%)	Hospital patients/outpatients	Asia	64 (47.4%)
Laskou	2022	641	69.3 ± 2.6	12	319 (49.8%)	Community	Europe	108 (16.8%)
Muraki	2012	2215	68.5 ± 11.3	36	1470 (66.4%)	Community	Asia	503 (22.7%)
Ooi	2021	1763	68.6 ± 6.0	18	896 (50.8%)	Community	Asia	309 (17.5%)
Reijnierse	2019	222	79.4 ± 6.0	3	113 (50.9%)	Hospital patients/outpatients	Europe	43 (19.4%)
Sayer	2006	2148	66.8 ± 2.7	12	1282 (59.7%)	Community	Europe	413 (19.2%)
Valenzuela	2020	24	88.0 ± 7.0	12	19 (79.2%)	Community	Europe	12 (50.0%)
Westbury	2020	2689	74.0 ± 2.9	12	1397 (52.0%)	Community	North America	1052 (39.1%)
Appendicular lean mass								
Reijnierse	2019	222	79.4 ± 6.0	3	113 (50.9%)	Hospital patients/outpatients	Europe	43 (19.4%)
Westbury	2020	2689	74.0 ± 2.9	12	1397 (52.0%)	Community	North America	1052 (39.1%)
Walking speed								
Adam	2021	2705	78.5 ± 3.2	18	1221 (45.1%)	Community	North America	487 (18.0%)
Beauchet	2008	213	84.4 ± 5.5	12	178 (83.6%)	Community	Europe	57 (26.8%)
Bergland	2003	307	≥ 65	12	307 (100.0%)	Community	Europe	155 (50.5%)
Blackwood	2021	34	72.6 ± 5.7	3	34 (100.0%)	Hospital patients/outpatients	North America	14 (41.2%)
Faulkner	2009	8378	71.0 ± 3.0	48	8378 (100.0%)	Community	North America	4995 (59.6%)
Laskou	2022	641	69.3 ± 2.6	12	319 (49.8%)	Community	Europe	108 (16.8%)
Makino	2021	2520	71.1 ± 4.7	12	1303 (51.7%)	Community	Asia	415 (16.5%)
Makizako	2013	42	75.6 ± 6.3	12	18 (42.9%)	Hospital patients/outpatients	Asia	11 (26.2%)
Morone	2014	64	67.4 ± 12.5	12	24 (37.5%)	Hospital patients/outpatients	Europe	32 (50.0%)
Muraki	2012	2215	68.5 ± 11.3	36	1470 (66.4%)	Community	Asia	503 (22.7%)
Parsons	2020	258	75.5 ± 2.6	12	129 (50.0%)	Community	Europe	70 (27.1%)
Scott	2014	135	76.7 ± 5.0	NA	135 (100.0%)	Community	Oceania	74 (54.8%)
Wada	2020	74	≥ 65	12	38 (60.7%)	Hospital patients/outpatients	Asia	24 (32.4%)
Westbury	2020	2689	74.0 ± 2.9	12	1397 (52.0%)	Community	North America	1052 (39.1%)

*N* sample size, *NA* not applicable, *SD* standard deviation

#### Study quality

Supplementary Table 7 shows the NOS quality assessment results for the included studies. Twenty [11, 12, 36, 38, 39, 42, 43, 46, 47, 50–53, 57–60, 63–65] were rated as high quality and 14 as low [37, 40, 41, 44, 45, 48, 49, 54–56, 61, 62, 66, 67].

#### Association of Sarcopenia-related traits and risk of falls

**Fig. 3:**
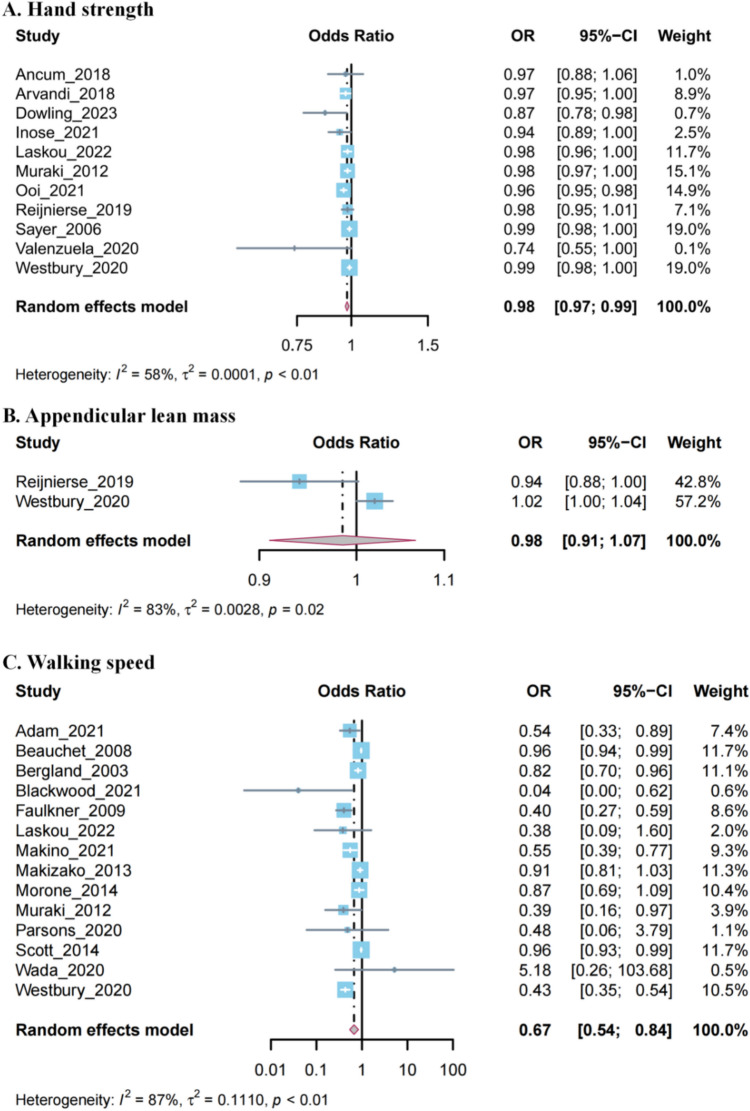
Forest plots of meta-analysis odds ratio for falls in continuous sarcopenia-related traits in cohort study. **A** Hand strength; **B** Appendicular lean mass; **C** Walking speed. Forest plot showing the individual and summary random effects estimates for associations between each 1-unit increase sarcopenia-related traits and falls in cohort study. The blue squares represent the weight of each study, and the gray bars show the 95% CIs. The grey and maroon diamond shows the pooled odds ratio

Among the studies we included, 22 studies presented sarcopenia-related traits as continuous variables [37–47, 53–63]. By pooling these studies, we found that each 1-unit increase in walking speed was associated with a 33% decrease in fall risk (OR 0.67, 95% CI 0.54–0.84), and each 1-unit increase in hand strength was associated with a 2% decrease in fall risk (OR 0.98, 95% CI 0.97–0.99). However, the effect of appendicular lean mass on falls did not reach statistical significance (OR 0.98, 95% CI 0.91–1.07) (Fig. 3). Heterogeneity was evident with I^2^ values exceeding 50% across all analyses. Similar trend was observed in the pooled analyses involving studies that presented sarcopenia-related traits as categorical variables (Supplementary Fig. 1).

#### Subgroup and sensitivity analyses

Subgroup analyses were conducted to investigate the source of the heterogeneity. The analysis revealed variations in fall risk related to walking speed between community participants and hospital patients (OR _Community_ 0.62 [95% CI 0.48–0.81] vs. OR _Patients_ 0.90 [95% CI 0.81–1.00]; *P* = 0.01), as well as between male and female (OR _Male_ 0.26 [95% CI 0.15–0.45] vs. OR _Female_ 0.64 [95% CI 0.44–0.94]; *P* < 0.01). The geographical location of the studies contributed to the diversity in fall risk (OR _North America_ 0.43 [95% CI 0.36–0.52] vs. OR _Europe_ 0.90 [95% CI 0.80–1.00] vs. OR _Asia_ 0.69 [95% CI 0.44–1.07] vs. OR _Oceania_ 0.96 [95% CI 0.93–0.99]; *P* < 0.01). Subgroup analyses based on population, continent, follow-up time, study quality, sex, and walking test distance were generally consistent with the primary analysis, affirming the robustness of our findings (Supplementary Figs. 2–5). The findings remained consistent in the leave-one-out analysis (Supplementary Fig. 6).

#### Publication bias

The publication bias was assessed through the visual examination of funnel plots (Supplementary Fig. 7) and the Egger weighted regression analysis (*P* = 0.012 and 0.005 for hand strength and walking speed), revealing indications of partial publication bias. Since only two appendicular lean mass studies were included in our meta-analysis, no Egger test was conducted. Trimming and filling analysis indicated only 5 missing studies were required to achieve a symmetrical funnel plot for both hand strength and walking speed studies.

### Mendelian randomization

#### Selection of instrumental variables

We selected 125 SNPs for hand strength, 616 SNPs for appendicular lean mass, and 45 SNPs for walking speed as IVs. The F-statistic for all IVs was greater than 10 (Supplementary Table 9).

#### MR Analysis

**Fig. 4:**
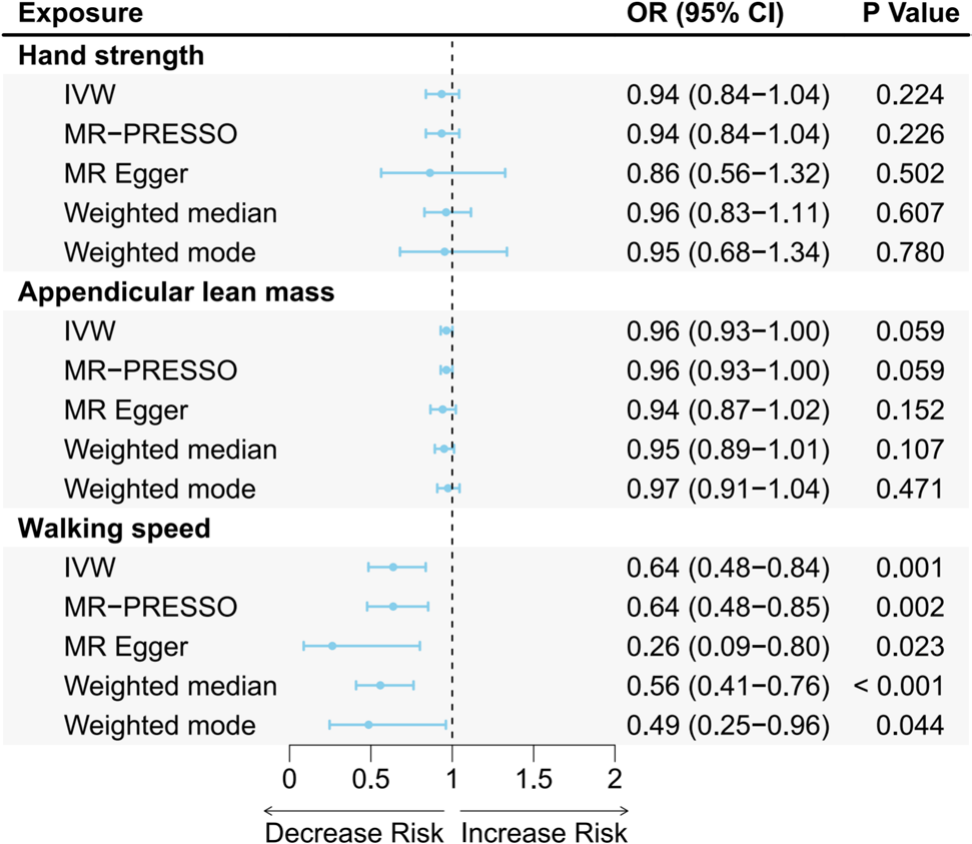
Forest plot for summary causal effects of sarcopenia-related traits on fall risk based on five MR methods. Forest plot showing the associations between sarcopenia-related traits and falls estimated by the MR methods. The blue round represents the point estimates of the OR, and the blue bars show the 95% CIs. *MR* Mendelian randomization, *IVW* inverse variance weighted, *OR* odds ratio, *CIs* confidence intervals

We found that genetically predicted walking speed was causally associated with a reduced risk of falls (IVW _walking speed_: OR 0.64, 95% CI 0.48–0.84, *P* = 0.001). However, there were no statistically significant associations between hand strength and appendicular lean mass for falls (IVW _hand strength_: OR 0.94, 95% CI 0.84–1.04, *P* = 0.224; IVW _appendicular lean mass_: OR 0.96, 95% CI 0.93–1.00, *P* = 0.059) (Fig. 4). Consistent results were obtained for sarcopenia-related traits with falls using alternative MR methods (Supplementary Fig. 9 and Supplementary Table 10).

Significant heterogeneity was detected in all MR analyses based on Cochran’s Q test (*P*_hand strength_ = 0.0108; *P*_appendicular lean mass_ = 0.0003; *P*_walking speed_ = 0.0019), and we utilized the random effects IVW model for the analyses. The MR Egger intercept test, conducted to assess horizontal pleiotropy, showed no significant presence of horizontal pleiotropy (*P*_hand strength_ = 0.71, *P*_appendicular lean mass_ = 0.52, *P*_walking speed_ = 0.12) (Supplementary Table 11). The inferred causal direction was further confirmed through the MR Steiger test.

## Discussion

### Main findings

Sarcopenia is recognized as a modifiable risk factor for falls and fractures in older adults, while the relationship between sarcopenia-related traits (including hand strength, appendicular lean mass, and walking speed) and falls in older adults needs to be confirmed. In this study, we conducted a meta-analysis triangulated with two-sample MR analysis to consolidate cohort evidence and assess the causality between sarcopenia-related characteristics and the risk of falls in older adults. The results of the meta-analysis showed that each 1-unit increase in walking speed was associated with a 33% reduction in the risk of falls (OR 0.67, 95% CI 0.54–0.84) and each 1-unit increase in hand strength was associated with a 2% decrease in risk of falls (OR 0.98, 95% CI 0.97–0.99). The appendicular lean mass did not exhibit a statistically significant effect on fall risk (OR 0.98, 95% CI 0.91–1.07). However, the results of MR analyses only supported the causal effect of walking speed on fall risk, indicating that walking speed is a reliable predictor of fall risk, whereas the relationship between hand strength, appendicular lean mass, and falls need to be further investigated. To visualize the effect of changes in sarcopenia-related characteristics on fall risk, we plotted probability curves based on meta-analysis and MR results (Supplementary Fig. 10).

### Comparison with other studies

In the present study, we revealed a negative relationship between walking speed and fall risk through a meta-analysis of 14 cohort studies and further confirmed a causal relationship using MR. This result was consistent with previous studies that explored the relationship between walking speed and falls. A previously published systematic review of 17 retrospective studies identified that almost all of them found that non-fall participants had higher walking speeds than those who had fallen [68]. A more recent systematic review including 21 cross-sectional studies and 19 cohort studies published between 2006 and 2019, indicated that most studies showed an increased proportion of slow gait in participants who had experienced recent falls [69].

Hand strength is a basic indicator for assessing muscle function and overall fitness status and is particularly relevant to the aging population [70, 71]. In our study, the meta-analysis found that each 1-unit increase in hand strength was associated with a 2% decrease in fall risk. A meta-analysis of 13 prospective studies reported that upper limb weakness is a significant risk factor for falls (OR 1.53, 95% CI 1.01–1.59), as is lower limb weakness (OR 1.76, 95% CI 1.31–2.37) [72]. Another systematic review that included 20 studies found a low but statistically significant relationship between trunk muscle strength and trunk muscle attenuation and falls in older adults [73]. However, a recent systematic review published in 2021 indicated there was no evidence to support an association between muscle strength and falls [74], which was similar to the results of our MR analysis. The discrepancy between the meta-analysis and MR results may be attributed to different participant ages in respective studies. The meta-analysis included individuals aged 65 years or older, whereas the MR study did not restrict the age of participants. This difference may have influenced the results since older people are at a higher risk of falling. Additionally, as with other observational studies, meta-analysis of cohort studies cannot completely exclude measurement errors and residual confounding. Moreover, accidental falls in the meta-analysis represent follow-up outcomes over a period of time, whereas MR assesses the life-long effects of genetic variation. Thus, larger longitudinal studies of longer duration and more representative GWAS data are needed to further validate the relationship between hand strength and fall risk.

Appendicular lean mass is a central parameter that defines sarcopenia, but its relationship with fall risk remains uncertain [75]. We did not find a significant association between appendicular lean mass and falls in older adults during the meta-analysis of five included studies, and similar results were observed in the MR analysis. Currently, there is a lack of comprehensive reviews on the association between appendicular lean mass and falls. Although our result is consistent with the majority of previous observational studies, due to the limited number of studies included in the meta-analysis and the results of the MR analysis being borderline significant (*P* = 0.059), our conclusion should be interpreted with caution.

### Clinical implication

Walking speed is not only a key indicator of a person’s ability to ambulate but also a reflection of their overall health, including muscle strength, balance, neural control, and cognitive function. Our meta-analysis and MR study indicated that walking speed is a significant risk factor for falls in older adults. This finding could potentially assist clinicians in identifying individuals at high risk of falls among older adults through simple body measurements.

Given that walking speed is a potentially modifiable factor, our study could serve as a motivation for clinical trials to explore whether improving walking speed through functional exercise or pharmacotherapy could reduce the incidence of falls among older adults. At present, we recommend individualized training programs tailored to each patient’s specific needs. For instance, patients with slower walking speeds should focus on speed enhancement training, while those with inadequate muscle strength should prioritize strength-building exercises.

In addition, regular assessment of walking speed and other relevant indicators is essential for monitoring progress and making timely adjustments to the training program. This personalized, adaptive approach ensures that interventions are consistently effective and ultimately lead to optimal training outcomes.

However, the relationship between hand strength and appendicular lean mass with falls remains unclear and needs to be further studied.

### Potential mechanism

Walking speed serves as an indicator of a person’s walking ability and often reflects their overall health, including aspects of muscle strength, balance, neural control, and cognitive function. The relationship between walking speed and falls is influenced by a complex interplay of physiological, neurological, and environmental factors.

A slower walking speed can indicate declines in muscle strength and balance, both critical for fall prevention [76]. Reduced muscle strength diminishes walking stability and speed, while poor balance increases the risk of falling [77]. Furthermore, changes in walking speed may coincide with gait abnormalities, such as unsteady walking patterns or inconsistent stride lengths, which further increases the risk of falls.

Gait speed may also reflect the condition of the cardiovascular system. Cardiovascular disease can impair circulation, leading to reduced muscle oxygenation and nutrition, which in turn affects gait speed and stability and increases the risk of falls [78]. Recent studies have shown that high blood pressure, dyslipidemia, and the use of specific medications can damage microvessels in the brain, impairing an individual’s ability to walk and maintain balance [79].

Moreover, gait speed is related to neurological function, cognition, and psychological state. A slow gait may indicate prolonged reaction times, impairing an individual’s ability to respond effectively in emergency situations, thus increasing fall risk. Slower gait has also been linked to cognitive decline, including reduced attention and memory [80]. Declining cognitive function can affect decision-making and attention allocation during walking, thereby raising the risk of falls [81, 82]. Additionally, the use of psychotropic medications may contribute to slower gait speeds, further increasing fall risk [83].

In summary, walking speed not only reflects walking ability but is also closely related to overall health, muscle strength, balance, neural control, and cognitive function. A slower walking speed may be the result of a combination of multiple physiological, neurological, and environmental factors, all of which are associated with an increased risk of falls. Therefore, monitoring and improving gait speed is critical to preventing falls in older adults.

### Further research

Currently, the relationship between hand strength, appendicular lean mass, and falls remains ill-defined due to limitations in sample size, follow-up duration, and the number of studies. Larger and longer follow-up studies are necessary to clarify these relationships. Additionally, subgroup analyses reveal significant differences among populations, sexes, and continents. Therefore, it is essential to conduct large-scale prospective studies across various populations, sexes, and regions. Furthermore, previous research on walking speed has primarily focused on the relationship between optimal capacity and fall risk. Future studies are necessary to explore whether maximal capacity has a similar effect. Moreover, further investigation into the potential mechanisms linking sarcopenia-related traits to falls is imperative. Lastly, more interventional studies are needed to determine whether improvement of sarcopenia-related traits through exercise or pharmacotherapy is effective in reducing the incidence of falls.

### Strengthens and limitations

The main strength of our study lies in the precise estimation of the association between sarcopenia-related traits and risk of falls among older adults through two robust, evidence-based methods, meta-analysis and MR, which validate each other and increase the credibility of the results. We also performed a series of subgroups and sensitivity analyses, and the results were consistent with the main findings. However, our study also has some limitations. Firstly, despite conducting a comprehensive literature search on sarcopenia-related traits and falls from PubMed, Embase, and Cochrane Library, there was still a partial publication bias, which may have been due to some unpublished negative results. Secondly, although we performed subgroup analyses to explore the sources of heterogeneity in the study, we could not fully explain the heterogeneity present in the analyses. Thus, caution should be exercised in interpreting the results. Thirdly, the different studies included in the meta-analysis may select different confounders for adjustment based on their study design and data availability, which may introduce bias in effect estimates and influence the results of meta-analysis. To minimize this effect, we prioritized the use of ORs from fully adjusted models for pooling to obtain the most reliable results across studies and considered covariate adjustment when assessing study quality. Fourthly, previous studies on walking speed have primarily focused on the relationship between optimal capacity and fall risk. Our meta-analysis has also confirmed this association, but it remains unclear whether maximal capacity has a similar effect. Fifthly, since the GWAS used in the MR were obtained from public databases, we are unable to perform separate analyses specifically for older people to more accurately determine the causal relationship between sarcopenia-related traits and falls in this group. Lastly, given the unavailability of data stratified by ethnicity, subgroup analyses were conducted by continent rather than ethnicity. Our MR analysis was performed in European populations and therefore caution should be exercised when extrapolating to other ethnic populations.

## Conclusion

Our meta-analysis and MR study suggest a robust causal link between walking speed and fall risk in older adults. However, the association between hand strength, appendicular lean mass, and falls has not yet been established. Therefore, walking speed can be considered a more reliable indicator for predicting falls in older adults. Well-designed large longitudinal cohort studies and more representative GWAS data are required to further validate the associations between hand strength, appendicular lean mass, and fall risks in older adults.

## Supplementary Information

Below is the link to the electronic supplementary material.

Supplementary file1 (PDF 4657 kb)

## Data Availability

No datasets were generated or analysed during the current study.
