# Supplementary material for: Sarcopenia-related traits and risk of falls in older adults: results from meta-analysis of cohort studies and Mendelian randomization analyses
Source: Aging Clin Exp Res. 2025 Mar 26;37(1):106. doi: 10.1007/s40520-025-02997-7 (PMC11946949; doi:10.1007/s40520-025-02997-7)
Supplement: Supplementary file 1 — Supplementary file1 (PDF 4657 kb) [file 40520_2025_2997_MOESM1_ESM.pdf]

Supplementary File: Search Strategy

Supplementary Table 1. Systematic search strategy used for Pubmed

database	search strategy	N
Pubmed	#1 ((falls[Title/Abstract]) OR (faller*[Title/Abstract])) OR ("Accidental Falls"[Mesh])	67,574
	#2 "Hand Strength"[MeSH Terms] OR ("Muscle Strength Dynamometer"[MeSH Terms] AND "Muscle Strength"[MeSH Terms]) OR ("Muscle Strength"[Title/Abstract] OR "Hand Strength"[Title/Abstract] OR "handgrip"[Title/Abstract])	50,608
	#3 (ALM[Title/Abstract]) OR (appendicular lean mass[Title/Abstract])	1,650
	#4 (((walking speed[Title/Abstract])) OR (walking pace[Title/Abstract])) OR ("Walking Speed"[Mesh])	9,881
	#5 #1 and #2	1,739
	#6 #1 and #3	60
	#7 #1 and #4	931
	#8 "Review" [Publication Type]	3,111,971
	#9 "Editorial" [Publication Type]	635,794
	#10 "Letter" [Publication Type]	1,206,902
	#11 “Case Reports” [Publication Type]	2,317,782
	#12 #5 NOT #8 NOT #9 NOT #10 NOT #11 Filters: Human	1,272
	#13 #6 NOT #8 NOT #9 NOT #10 NOT #11 Filters: Humans	47
	#14 #7 NOT #8 NOT #9 NOT #10 NOT #11 Filters: Humans	785

Supplementary Table 2. Systematic search strategy used for Embase

database	search strategy	N
Embase	#1 faller:ab,ti OR falls:ab,ti OR 'falling'/exp	105,597
	#2 'hand strength':ab,ti OR handgrip:ab,ti OR 'muscle strength':ab,ti OR 'hand strength'/exp	77,117
	#3 alm:ab,ti OR 'appendicular lean mass':ab,ti	2,593
	#4 'walking pace':ab,ti OR 'walking speed':ab,ti OR 'walking speed'/exp	25,180
	#5 #1 AND #2 AND [article]/lim AND [humans]/lim AND [clinical study]/lim	1,280
	#6 #1 AND #3 AND [article]/lim AND [humans]/lim AND [clinical study]/lim	45
	#7 #1 AND #4 AND [article]/lim AND [humans]/lim AND [clinical study]/lim	1,209

Supplementary Table 3. Systematic search strategy used for Cochrane Library

database	search strategy	N
Cochrane Library	#1 (faller):ti,ab,kw OR (falls):ti,ab,kw (Word variations have been searched)	23,347
	#2 MeSH descriptor: [Accidental Falls] explode all trees	1,919
	#3 (hand strength):ti,ab,kw OR (handgrip):ti,ab,kw OR (muscle strength):ti,ab,kw (Word variations have been searched)	30,123
	#4 MeSH descriptor: [Hand Strength] explode all trees	2,000
	#5 (ALM):ti,ab,kw OR (appendicular lean mass):ti,ab,kw (Word variations have been searched)	356
	#6 (walking pace):ti,ab,kw OR (walking speed):ti,ab,kw (Word variations have been searched)	7,065
	#7 MeSH descriptor: [Walking Speed] explode all trees	267
	#8 #1 OR #2	23,347

	#9 #3 OR #4	30,129
	#10 #6 OR #7	7,065
	#11 #8 AND #9 in Cochrane Reviews, Cochrane Protocols, Trials	1,714
	#12 #8 AND #5 in Cochrane Reviews, Cochrane Protocols, Trials	33
	#13 #8 AND #10 in Cochrane Reviews, Cochrane Protocols, Trials	962

Supplementary Table 4. Systematic search strategy of Sarcopenia and its association with falls used for Pubmed, Embase and Cochrane Library

database	search strategy	N
Pubmed	#1 ("Sarcopenia"[Mesh]) AND ("Accidental Falls"[Mesh])	199
	#2 ((("Review" [Publication Type]) OR ("Editorial" [Publication Type])) OR ("Letter" [Publication Type])) OR ("Case Reports" [Publication Type])	6,928,010
	#3 #1 NOT #2 Filters: Humans	139
Embase	#1 'sarcopenia'/exp	18,956
	#2 'falling'/exp	49,125
	#3 #1 AND #2 AND [article]/lim AND [humans]/lim AND [clinical study]/lim	142
Cochrane Library	#1 MeSH descriptor: [Sarcopenia] explode all trees	802
	#2 MeSH descriptor: [Accidental Falls] explode all trees	1,921
	#3 #1 and #2 in Cochrane Reviews, Cochrane Protocols, Trials	13

Supplementary File: Study characteristic

Supplementary Table 5. Study characteristics of sarcopenia-related traits as categorical variables and falls outcomes

Author	Year	N	Mean/Median age ± SD (years)	Follow-up time (months)	Female, n (%)	Population	Continent	Falls incidence, n (%)
**Hand strength**								
Bath	1998	444	≥65	12	241 (54.3%)	Community	Europe	117 (26.4%)
Cawthon	2020	15103	≥65	NA	4828 (26.5%)	Community	Global	3020 (20.0%)
Dowling	2023	4239	69.4±6.6	24	2279 (53.8%)	Community	Europe	1049 (24.7%)
Lin	2017	70	86.1±4.0	NA	NA	Hospital patients/outpatients	Asia	52 (74.3%)
Pluijm	2006	1365	75.3±6.4	36	698 (51.1%)	Community	Europe	337 (24.7%)
Schaap	2018	496	75.2±6.4	36	250 (50.4%)	Community	Europe	130 (26.2%)
Sjoblom	2013	548	67.9±1.9	12	548 (100%)	Community	Europe	119 (21.7%)
Zhang	2022	4987	≥65	168	1576 (31.6%)	Community	Europe	1576 (31.6%)
**Appendicular lean mass**								
Cawthon	2020	15103	≥65	NA	4828 (26.5%)	Community	Global	3020 (20.0%)
Ferrari	2015	445	70.9±4.6	36	246 (55.3%)	Community	North America	231 (51.9%)
Schaap	2018	496	75.2±6.4	36	250 (50.4%)	Community	Europe	130 (26.2%)
**Walking speed**								
Abolhassani	2022	1887	74.9±1.4	12	1128 (59.8%)	Community	Europe	399 (21.1%)
Bath	1998	444	≥65	12	241 (54.3%)	Community	Europe	117 (26.4%)
Marini	2022	328	70.0±7.0	12	161 (49.1%)	Community	Europe	22 (6.7%)
Pua	2019	323	77.8±7.1	6	248 (76.8%)	Community	Asia	80 (24.8%)
Sayer	2006	2148	66.8±2.7	12	1282 (59.7%)	Community	Europe	413 (19.2%)
Schaap	2018	496	75.2±6.4	36	250 (50.4%)	Community	Europe	130 (26.2%)
Sjoblom	2013	548	67.9±1.9	12	548 (100%)	Community	Europe	119 (21.7%)
Stenhagen	2013	1542	≥65	72	826 (53.6%)	Community	Europe	205 (13.8%)

N, sample size; NA, not applicable; SD, standard deviation.

Supplementary File: Study-adjusted covariates

Supplementary Table 6. Study-adjusted covariates of sarcopenia-related traits and falls

Author	Year	Study-adjusted covariates
**Hand Strength**		
Ancum	2018	age, comorbidities and height
Arvandi	2018	age and sex
Bath	1998	-
Cawthon	2020	age, self-rated health,pain, use of statins, cognitive function, cancer, congestive heart failure, stroke, chronic obstructive pulmonary disease, and diabetes
Dowling	2023	age and sex
Inose	2021	age,sex and C-JOA score
Laskou	2022	age, BMI, social class, smoker status, alcohol consumption, physical activity (ascertained from 1998 to 2004), dietary calcium intake (ascertained from 1998–2004), hormone replacement therapy use (females only), time since menopause (females only), use of bisphosphonates and use of medications for the endocrine system
Lin	2017	age, education, tobacco smoking, alcohol consumption, sleep disorders, continence issues, polypharmacy, hypertension, coronary artery disease, stroke, diabetes mellitus, hyperlipidemia, and chronic obstructive pulmonary disease
Muraki	2012	age and BMI
Ooi	2021	age, sex, multimorbidity, falls history at baseline, years of education, and cognitive performance
Pluijm	2006	two or more previous falls, dizziness,functional limitations, weak grip strength, low body weight,fear of falling, the presence of dogs/cats in the household, ahigh education level, the drinking of 18 or more alcoholic consumptions per week
Reijnierse	2019	-
Sayer	2006	age,BMI, grip strength, height and walking speed
Schaap	2018	age, sex and total body fat mass
Sjoblom	2013	age, body mass index (BMI), physical activity and hormone therapy (HT), consumption of alcohol and smoking
Valenzuela	2020	age,gender, body mass index, and previous history of falls
Westbury	2020	age ,sex, ethnicity, height, weight-for-height residual (not used in models for level relating to ALM and fat mass), smoking status (ever vs never), alcohol consumption, healthy eating index, physical activity, educational attainment, home ownership, cognitive function, and number of comorbidities
Zhang	2022	age, sex, BMI, number of falls before baseline, smoking, alcohol drinking, physical activity, vision, hearing, depressive symptoms, cognition, dizziness, diabetes, stroke, arthritis, osteoporosis.
**Appendicular Lean Mass**		
Cawthon	2019	age, self-rated health,pain, use of statins, cognitive function, cancer, congestive heart failure, stroke, chronic obstructive pulmonary disease, and diabetes.
Ferrari	2015	age, treatment, gender, BMI, and baseline serum 25(OH)D level.
Reijnierse	2019	-
Schaap	2018	age, sex, total body fat and the other sarcopenia components

Westbury	2021	age ,sex, ethnicity, height, weight-for-height residual (not used in models for level relating to ALM and fat mass), smoking status (ever vs never), alcohol consumption, healthy eating index, physical activity, educational attainment, home ownership, cognitive function, and number of comorbidities
**Walking Speed**		
Abolhassani	2022	age, sex, and respective values of each adverse outcome
Adam	2021	age, sex, treatment arm, study site, and MCI
Bath	1998	-
Beauchet	2008	-
Bergland	2003	-
Blackwood	2021	Functional Comorbidity Index
Faulkner	2009	age, fall history at baseline, clinic, waist-to-hip circumference, stroke, Parkinson's disease, diabetes, arthritis, self-rated health, standing balance with eyes open, rapid stepping, grip strength, alcohol consumption, hours per day spent on feet, hours per week doing household chores, dizziness upon standing, fear of falling, visual acuity decline, fall history at baseline, use of benzodiazepines, use of antidepressants, use of antiepileptics, number of IADLs with difficulty, standing balance with eyes closed, usual walking speed, smoking status, physical activity, and frequency of going outdoors.
Laskou	2022	age, BMI, social class, smoker status, alcohol consumption, physical activity (ascertained from 1998 to 2004), dietary calcium intake (ascertained from 1998–2004), hormone replacement therapy use (females only), time since menopause (females only), use of bisphosphonates and use of medications for the endocrine system
Makino	2021	age, fall history, fear of falling, polypharmacy, knee osteoarthritis, and lower limb pain
Makizako	2013	age, sex, body mass index (kg/m 2) and history of falling in the past year at baseline
Marini	2022	age and sex
Morone	2014	age, Barthel Index, WS6, WS6/WS10
Muraki	2012	age, BMI, walking speed, radiographic knee OA, and knee pain as independent variable
Parsons	2020	age,sex and height
Pua	2019	randomization group assignment, sex, age, ethnicity, a history of stroke or Parkinson's disease, falls history, vision, bodily pain, Modified Falls Efficacy Scale score, Montreal Cognitive Assessment score, baseline self-reported mobility limitations, and the type of gait aids used
Sayer	2006	age, grip strength, height and walking speed
Schaap	2018	age, sex and total body fat mass
Scott	2014	age, BMI, years in study, vitamin D, falls and fracture history, antidepressant/anti-anxiety and non-steroidal anti-inflammatory (NSAID) medication use, and the baseline value for the relevant gait parameter
Sjoblom	2013	age, body mass index (BMI), physical activity and hormone therapy (HT), consumption of alcohol and smoking
Stenhagen	2013	age and sex
Wada	2020	age, sex, previous fall history, JOA score, HADS-depression score, muscle weakness (tibialis anterior), and low muscle mass
Westbury	2020	age ,sex, ethnicity, height, weight-for-height residual (not used in models for level relating to ALM and fat mass), smoking status (ever vs never), alcohol consumption, healthy eating index, physical activity, educational attainment, home ownership, cognitive function, and number of comorbidities

Supplementary File: Newcastle-Ottawa Scale quality assessment

Supplementary Table 7. Results of the Newcastle-Ottawa Scale quality assessment for cohort studies

Author	Year	Selection				Comparability	Outcome			Score	Quality
		Representativeness	Selection	Ascertainment	Outcome was not present at start of study	Adjustment for confounders	Assessment	Duration of follow up	Completeness of follow up		
**Hand Strength**											
Ancum	2018	-	*	*	*	**	-	-	*	6	Low
Arvandi	2018	*	*	*	*	*	-	*	*	7	High
Bath	1998	*	*	-	*	-	-	*	-	4	Low
Cawthon	2020	*	*	*	*	**	-	*	*	8	High
Dowling	2023	*	*	*	*	*	-	*	*	7	High
Inose	2021	-	*	-	*	**	*	*	-	6	Low
Laskou	2022	*	*	*	-	**	-	*	-	6	Low
Lin	2017	-	*	-	-	**	-	*	-	4	Low
Muraki	2012	*	*	*	*	**	-	*	-	7	High
Ooi	2021	*	*	*	-	**	-	*	*	7	High
Pluijm	2006	*	*	*	*	**	*	*	*	9	High
Reijnierse	2019	-	*	*	*	-	-	-	-	3	Low
Sayer	2006	*	*	-	-	**	-	*	*	6	Low
Schaap	2018	*	*	*	*	**	*	*	*	9	High
Sjoblom	2013	*	*	*	-	**	-	*	*	7	High
Valenzuela	2020	*	*	*	*	**	*	*	*	9	High
Westbury	2020	-	*	*	*	**	-	*	*	7	High
Zhang	2022	*	*	*	*	**	-	*	-	7	High
**Appendicular Lean Mass**											
Cawthon	2019	*	*	*	*	**	*	*	-	8	High
Ferrari	2015	*	*	*	*	**	-	*	-	7	High
Reijnierse	2019	-	*	*	*	-	-	-	-	3	Low
Schaap	2018	*	*	*	*	**	*	*	*	9	High
Westbury	2020	-	*	*	*	**	-	*	*	7	High
**Walking Speed**											
Abolhassani	2022	*	*	*	*	**	-	*	*	8	High
Adam	2021	*	*	*	*	**	-	*	*	8	High
Bath	1998	*	*	-	*	-	-	*	-	4	Low

Beauchet	2008	*	*	*	*	-	-	*	*	6	Low
Bergland	2003	*	*	-	*	-	-	*	*	5	Low
Blackwood	2021	*	*	*	*	*	-	-	*	6	Low
Faulkner	2009	*	*	*	*	**	-	*	*	8	High
Laskou	2022	*	*	*	-	**	-	*	-	6	Low
Makino	2021	*	*	*	*	**	-	*	*	8	High
Makizako	2013	-	*	*	*	**	-	*	*	7	High
Marini	2022	*	*	*	*	*	*	*	-	7	High
Morone	2014	-	*	*	*	**	-	*	*	7	High
Muraki	2012	*	*	*	*	**	-	*	-	7	High
Parsons	2020	*	*	*	-	**	-	*	-	6	Low
Pua	2019	*	*	*	*	**	-	-	-	6	Low
Sayer	2006	*	*	-	-	**	-	*	*	6	Low
Schaap	2018	*	*	*	*	**	*	*	*	9	High
Scott	2014	*	*	*	-	**	*	-	-	6	Low
Sjoblom	2013	*	*	*	-	**	-	*	*	7	High
Stenhagen	2013	*	*	*	*	*	-	*	-	6	Low
Wada	2020	-	*	*	*	**	*	*	*	8	High
Westbury	2020	-	*	*	*	**	-	*	*	7	High

A dash “-” is scored as 0, a single asterisk “*” is scored as 1, and a double asterisk “**” is scored as 2.

Supplementary File: Mendelian randomization supplementary table

Supplementary Table 8. Information of included studies and consortium

Exposure/Outcome	Consortium	Participants
Hand strength	UK Biobank	461,026
Appendicular lean mass	UK Biobank	450,243
Walking speed	UK Biobank	459,915
Fall	FinnGen	377,277

Supplementary Table 9. Single nucleotide polymorphisms used as instrumental variables for falls

Exposure	SNP	EA	NEA	EAF	Beta	SE	P value	F statistic
Hand strength	rs10097417	A	G	0.170904	-0.0132302	0.00197055	1.9E-11	45.07710505
Hand strength	rs10176878	T	C	0.190861	-0.0129478	0.0018962	8.6E-12	46.62531686
Hand strength	rs10205394	G	C	0.200728	-0.0113217	0.00185667	1.1E-09	37.18364511
Hand strength	rs10403906	G	A	0.476375	-0.0100323	0.00148665	1.5E-11	45.53880885
Hand strength	rs1044299	C	T	0.546046	0.01401	0.00149362	6.6E-21	87.98206398
Hand strength	rs10786706	C	T	0.465745	0.0100072	0.00148741	1.7E-11	45.26493291
Hand strength	rs10788958	C	G	0.644753	0.0141976	0.00156276	1E-19	82.53599958
Hand strength	rs10821939	G	A	0.573342	-0.00935422	0.00150339	4.9E-10	38.71417075
Hand strength	rs10831903	C	T	0.423383	0.00928662	0.00151154	8.1E-10	37.74628173
Hand strength	rs10934857	G	A	0.258913	0.00928675	0.00170182	0.000000048	29.77819561
Hand strength	rs10988217	A	G	0.603742	-0.00920657	0.00152911	1.7E-09	36.25069841
Hand strength	rs11002322	G	T	0.340459	-0.00998924	0.00157255	2.1E-10	40.35097984
Hand strength	rs11076004	G	A	0.418177	-0.0115356	0.00150909	2.1E-14	58.43165808
Hand strength	rs11111267	A	G	0.18106	0.0108359	0.00192886	0.000000019	31.55925038
Hand strength	rs11121542	G	A	0.122677	-0.0157601	0.00225916	3E-12	48.66559905
Hand strength	rs11125803	C	T	0.740516	0.0142851	0.00169673	3.8E-17	70.88252968
Hand strength	rs11168357	G	A	0.246008	-0.00962864	0.00172661	0.000000025	31.09850495
Hand strength	rs11204664	T	C	0.578725	-0.00865184	0.00150032	8.1E-09	33.25425918
Hand strength	rs11243202	T	C	0.486019	0.00972237	0.0014909	7E-11	42.52510319
Hand strength	rs113918482	A	G	0.222507	-0.0101011	0.00178515	0.000000015	32.01739756
Hand strength	rs116409670	C	T	0.079819	-0.0152418	0.00273867	0.000000026	30.9735671
Hand strength	rs11669079	A	T	0.705026	0.0109542	0.00163169	1.9E-11	45.0696457
Hand strength	rs116825011	G	A	0.016094	0.0327595	0.00595077	0.000000037	30.30583926
Hand strength	rs116922558	A	G	0.039916	-0.0215842	0.00385425	0.000000021	31.36102631
Hand strength	rs11769549	T	A	0.062372	0.0204956	0.00311228	4.5E-11	43.36726816
Hand strength	rs12316046	A	G	0.37795	-0.0174165	0.00152991	5E-30	129.5949617
Hand strength	rs12473732	C	T	0.486507	0.0109869	0.00148601	1.4E-13	54.66445158
Hand strength	rs12528131	A	G	0.488343	-0.00882788	0.00148565	2.8E-09	35.30839232
Hand strength	rs12533765	A	G	0.280054	-0.0092	0.00165199	0.000000026	31.01408283
Hand strength	rs12673062	G	A	0.215756	-0.0107691	0.00180808	2.6E-09	35.47493847
Hand strength	rs12889267	A	G	0.167084	-0.0137394	0.00198726	4.7E-12	47.79960071
Hand strength	rs12906830	T	C	0.601065	0.0108401	0.00151797	9.3E-13	50.99625875
Hand strength	rs12914702	G	A	0.300395	0.0109514	0.00169071	9.3E-11	41.95649079
Hand strength	rs13091492	A	G	0.372833	-0.00847903	0.00153407	0.000000033	30.54921856
Hand strength	rs13106087	T	C	0.829801	0.0116202	0.00197399	3.9E-09	34.65256807
Hand strength	rs13146142	T	C	0.158623	-0.0202031	0.00202888	2.3E-23	99.15655299
Hand strength	rs13227429	T	C	0.561343	-0.00858767	0.0014977	9.8E-09	32.87752761

Hand strength	rs13337177	G	T	0.180836	-0.0142799	0.00193621	1.6E-13	54.39306488
Hand strength	rs143002906	C	T	0.027829	0.0262281	0.00457421	9.8E-09	32.87756005
Hand strength	rs143384	A	G	0.404388	0.0209202	0.00151159	1.5E-43	191.5410042
Hand strength	rs1434095	T	C	0.875121	0.0140301	0.00225906	5.3E-10	38.57128586
Hand strength	rs1486925	T	C	0.314604	-0.0104734	0.00160211	6.3E-11	42.73550442
Hand strength	rs150330307	T	C	0.031896	-0.0307753	0.0042167	2.9E-13	53.26688476
Hand strength	rs1556659	C	T	0.38153	0.0162896	0.00153452	2.5E-26	112.6870085
Hand strength	rs1641457	T	G	0.223305	0.0120456	0.0017829	1.4E-11	45.64582078
Hand strength	rs16870531	C	T	0.238492	0.011198	0.00174241	1.3E-10	41.3026895
Hand strength	rs16910750	G	C	0.159639	0.0112498	0.00204139	0.000000036	30.36936793
Hand strength	rs17282763	T	C	0.296281	0.00893956	0.00163298	0.000000044	29.96875296
Hand strength	rs17466480	A	G	0.386968	-0.0118235	0.00152732	9.8E-15	59.92805046
Hand strength	rs17630248	T	C	0.347808	-0.009184	0.00156162	4.1E-09	34.58686041
Hand strength	rs181766	T	C	0.321793	0.00963247	0.00160372	1.9E-09	36.07583197
Hand strength	rs1884447	G	A	0.400566	0.00846069	0.00151391	0.000000023	31.23270262
Hand strength	rs1981612	C	A	0.45601	0.00922755	0.00151053	0.000000001	37.31747239
Hand strength	rs2038760	C	T	0.170908	-0.0115262	0.00198662	6.6E-09	33.66206943
Hand strength	rs217181	C	T	0.193087	0.0119554	0.00188145	2.1E-10	40.3776441
Hand strength	rs2359239	C	T	0.391621	-0.00881461	0.00152153	6.9E-09	33.56164815
Hand strength	rs2532111	A	G	0.639542	0.0102852	0.00155794	4.1E-11	43.58350389
Hand strength	rs2587505	T	C	0.419871	-0.00900424	0.00150694	2.3E-09	35.702639
Hand strength	rs2631360	G	A	0.519209	-0.0109177	0.00148434	1.9E-13	54.09954928
Hand strength	rs2800789	A	C	0.480339	0.00826025	0.00149261	0.000000031	30.6261077
Hand strength	rs28542042	C	T	0.308877	0.0110101	0.00162277	1.2E-11	46.03272687
Hand strength	rs2871865	C	G	0.116247	-0.0217979	0.00231659	5E-21	88.53787069
Hand strength	rs2871960	A	C	0.444681	0.0120879	0.00149222	5.5E-16	65.6196818
Hand strength	rs2876637	C	T	0.198122	0.0103043	0.00186519	0.000000033	30.52031051
Hand strength	rs2974438	G	A	0.211026	-0.0100735	0.00182387	0.000000033	30.50500761
Hand strength	rs3118903	G	A	0.219552	-0.0174477	0.00179692	2.7E-22	94.27944159
Hand strength	rs34030812	T	C	0.367365	-0.0101679	0.00154042	4.1E-11	43.56947746
Hand strength	rs34587452	G	C	0.21517	-0.0113642	0.00180741	3.2E-10	39.53324746
Hand strength	rs34845616	G	A	0.245653	0.0108395	0.00173373	4E-10	39.08892141
Hand strength	rs35236379	G	T	0.142324	0.0123565	0.00212204	5.8E-09	33.9064195
Hand strength	rs35609019	G	C	0.397748	0.00947689	0.00154061	7.7E-10	37.83941124
Hand strength	rs3819121	T	C	0.368945	0.0140963	0.00152872	2.9E-20	85.02614498
Hand strength	rs4121165	G	A	0.211447	-0.0114169	0.00181868	3.4E-10	39.40777623
Hand strength	rs41271299	C	T	0.051241	0.0211891	0.00336402	3E-10	39.6740009
Hand strength	rs4308051	T	G	0.789105	0.0159929	0.00181863	1.4E-18	77.33282182
Hand strength	rs4335354	C	A	0.315536	-0.00939409	0.00160262	4.6E-09	34.35946815
Hand strength	rs4398863	G	C	0.7368	-0.00945015	0.00168454	0.00000002	31.47116821
Hand strength	rs4498020	C	A	0.723767	-0.0104238	0.00166995	4.3E-10	38.9621864
Hand strength	rs4575361	A	T	0.31205	-0.0108028	0.00160223	1.6E-11	45.45912469
Hand strength	rs4672335	G	A	0.147744	-0.0117733	0.00209025	0.000000018	31.7247401
Hand strength	rs4677601	A	G	0.510324	0.00905884	0.001485	1.1E-09	37.21263188
Hand strength	rs4737446	G	T	0.694626	0.0104178	0.00161882	1.2E-10	41.41455742
Hand strength	rs4739739	A	G	0.415057	-0.00853387	0.00150466	0.000000014	32.16721116
Hand strength	rs4811040	C	G	0.276636	-0.00924973	0.00166896	0.00000003	30.71597968
Hand strength	rs4930582	C	T	0.835661	0.011902	0.00202011	3.8E-09	34.7126661
Hand strength	rs4962700	C	G	0.301977	0.00904307	0.00163488	0.000000032	30.59554116

Hand strength	rs55681913	T	C	0.106095	0.0137738	0.00243658	0.000000016	31.95540388
Hand strength	rs56338231	A	G	0.258266	-0.010847	0.00169739	1.7E-10	40.83702705
Hand strength	rs58670122	A	G	0.143159	-0.0118148	0.00214462	0.000000036	30.3494036
Hand strength	rs59116179	C	T	0.617442	0.00857385	0.00153635	0.000000024	31.14365279
Hand strength	rs6006984	T	C	0.277764	0.00984917	0.00165413	2.6E-09	35.45341763
Hand strength	rs61389091	C	T	0.04174	0.0261627	0.00373295	2.4E-12	49.12006047
Hand strength	rs61818100	T	C	0.116595	0.0134422	0.00231335	6.2E-09	33.7641742
Hand strength	rs62081464	C	T	0.227338	-0.0098827	0.00177979	0.000000028	30.83271834
Hand strength	rs62253653	A	G	0.295379	0.0106985	0.00163146	5.5E-11	43.00223592
Hand strength	rs635538	G	A	0.913853	-0.0216569	0.00265852	3.8E-16	66.36068108
Hand strength	rs6680160	A	G	0.628066	0.0100686	0.00153878	6E-11	42.81374573
Hand strength	rs6802071	C	T	0.434928	-0.00940386	0.00150182	3.8E-10	39.20799721
Hand strength	rs6882168	C	T	0.337417	-0.00935387	0.00157364	2.8E-09	35.33214491
Hand strength	rs6962338	A	G	0.043993	-0.0214095	0.0036171	3.2E-09	35.03403297
Hand strength	rs6977081	G	T	0.333835	0.0147525	0.00158641	1.4E-20	86.47657588
Hand strength	rs7026798	T	C	0.43232	0.00823598	0.00150641	0.000000046	29.89112788
Hand strength	rs7148603	G	A	0.359427	0.00957206	0.00158612	1.6E-09	36.4197414
Hand strength	rs7176095	A	G	0.128192	-0.013375	0.00222025	1.7E-09	36.28957983
Hand strength	rs7196917	A	G	0.429948	-0.0117302	0.00150067	5.4E-15	61.09962553
Hand strength	rs7197751	G	T	0.363019	-0.00946321	0.00156268	1.4E-09	36.67203105
Hand strength	rs723588	T	C	0.142598	0.0127904	0.00211948	1.6E-09	36.4172983
Hand strength	rs72977282	T	A	0.413856	-0.0155569	0.00151173	7.8E-25	105.8999543
Hand strength	rs7516571	A	G	0.259017	0.00937798	0.00169361	0.000000031	30.66125321
Hand strength	rs75497896	T	C	0.051006	-0.0206727	0.00337524	9.1E-10	37.51312135
Hand strength	rs755547	G	A	0.188906	0.0165156	0.00189766	3.2E-18	75.74431128
Hand strength	rs7575451	C	G	0.649752	-0.00973095	0.00155349	3.8E-10	39.23662774
Hand strength	rs772014	A	G	0.392538	-0.0106178	0.00151891	2.7E-12	48.86558636
Hand strength	rs7963801	T	C	0.571927	-0.0104348	0.00150824	4.6E-12	47.86581588
Hand strength	rs7970350	C	T	0.494068	-0.0101345	0.00148418	8.6E-12	46.62615682
Hand strength	rs8108461	T	C	0.572775	0.00952393	0.0015066	2.6E-10	39.96083728
Hand strength	rs821100	A	G	0.265512	-0.0101977	0.00168615	1.5E-09	36.57717544
Hand strength	rs823130	C	T	0.432903	-0.0113317	0.00150098	4.4E-14	56.99522096
Hand strength	rs9371201	C	T	0.335102	-0.00933847	0.0015738	0.000000003	35.20874052
Hand strength	rs9371881	G	A	0.359174	0.00947946	0.00154996	9.6E-10	37.40454117
Hand strength	rs9388769	G	A	0.67318	-0.0140821	0.00158065	5.1E-19	79.37094163
Hand strength	rs9611273	C	T	0.25267	0.0107888	0.00173048	4.5E-10	38.86970387
Hand strength	rs9866627	C	A	0.084376	-0.0155989	0.00267761	5.7E-09	33.93840448
Hand strength	rs9944324	A	G	0.456723	-0.00855272	0.00150169	0.000000012	32.4374008
Hand strength	rs999493	G	A	0.621921	0.0128713	0.00153936	6.2E-17	69.91374191
ALM	rs1001506	A	G	0.2016	-0.0462	0.0024	9.78E-85	370.5608539
ALM	rs10028003	A	C	0.5189	-0.0191	0.0019	5.92E-24	101.0549528
ALM	rs1003167	G	T	0.3989	0.0233	0.0019	9.33E-34	150.3843735
ALM	rs1003834	G	A	0.0646	0.0214	0.0039	0.000000035	30.10900498
ALM	rs10065338	A	G	0.7835	-0.0146	0.0023	1.7E-10	40.29471704
ALM	rs10145110	C	T	0.3688	-0.0177	0.002	1.66E-19	78.32215209
ALM	rs10211504	G	T	0.2657	0.0129	0.0021	1.38E-09	37.73452626
ALM	rs10411903	A	G	0.3519	0.0137	0.002	5.77E-12	46.92229157
ALM	rs10429489	G	A	0.4157	0.0138	0.0019	6.85E-13	52.75322827
ALM	rs10503723	G	A	0.1208	-0.0175	0.0029	1.67E-09	36.41482041

ALM	rs10509391	G	A	0.2908	0.0121	0.0021	1.62E-08	33.19939901
ALM	rs10509953	T	C	0.3489	0.0114	0.002	9.53E-09	32.48985568
ALM	rs1072585	A	C	0.6816	0.0162	0.002	8.01E-16	65.60970856
ALM	rs10748593	C	T	0.0507	0.0322	0.0043	7.38E-14	56.07546751
ALM	rs10769111	T	G	0.3484	-0.0237	0.002	8.96E-33	140.4218762
ALM	rs10771262	A	G	0.3104	-0.0185	0.0021	1.76E-19	77.60736501
ALM	rs10824335	C	A	0.3419	-0.0158	0.002	3.56E-15	62.40972277
ALM	rs10840024	G	A	0.4076	-0.0272	0.0019	3.25E-45	204.9409179
ALM	rs10878028	C	T	0.4413	0.03	0.0021	1.74E-46	204.0807261
ALM	rs10897509	C	T	0.0825	-0.0442	0.0035	1.73E-37	159.4801079
ALM	rs1092027	A	G	0.9	-0.0333	0.0032	6.34E-26	108.289558
ALM	rs10949418	G	A	0.2586	0.0129	0.0023	2.19E-08	31.45732718
ALM	rs10956056	T	C	0.6813	0.016	0.002	3.55E-15	63.99971571
ALM	rs10978861	A	G	0.0507	0.0236	0.0043	4.79E-08	30.12209443
ALM	rs11048859	T	A	0.2318	0.0178	0.0022	1.43E-15	65.46251913
ALM	rs11055063	C	T	0.3867	-0.0113	0.0019	4.86E-09	35.37103402
ALM	rs11065208	G	A	0.7875	-0.0265	0.0023	2.02E-30	132.7498829
ALM	rs11071442	A	G	0.5826	0.023	0.0019	4.38E-33	146.5367452
ALM	rs11103650	G	A	0.4564	0.0106	0.0019	2.31E-08	31.12451548
ALM	rs11109859	C	A	0.4273	-0.0181	0.0019	2.1E-21	90.7502894
ALM	rs11119801	G	A	0.3872	0.0137	0.002	2.96E-12	46.92229157
ALM	rs11121179	G	T	0.3391	-0.0162	0.002	2.04E-16	65.60970856
ALM	rs11138745	A	G	0.2612	0.0135	0.0022	3.91E-10	37.65479141
ALM	rs111474958	C	T	0.6266	-0.0133	0.002	8.89E-11	44.22230356
ALM	rs111491620	C	T	0.4562	0.0203	0.0019	1.36E-26	114.1518475
ALM	rs111498409	G	A	0.3457	0.0147	0.002	1.22E-13	54.02226003
ALM	rs111544000	G	T	0.4197	-0.0173	0.0019	1.33E-19	82.9054489
ALM	rs11166137	T	A	0.7873	0.013	0.0023	0.000000021	31.94692803
ALM	rs111737303	C	T	0.13	-0.0205	0.0028	2.98E-13	53.60307822
ALM	rs111842294	C	T	0.0651	-0.0295	0.0038	1.57E-14	60.26635279
ALM	rs11190327	G	A	0.3496	-0.02	0.002	7.05E-24	99.9995558
ALM	rs112000670	T	C	0.6626	0.0159	0.002	1.65E-15	63.20221925
ALM	rs11217609	C	A	0.715	-0.0149	0.0021	1.49E-12	50.34218
ALM	rs112411507	A	G	0.2228	0.0282	0.0022	4.85E-36	164.3050553
ALM	rs11257078	A	G	0.2765	0.0122	0.0021	8.29E-09	33.75041697
ALM	rs112578789	A	G	0.4162	-0.0159	0.0019	1.15E-16	70.03015984
ALM	rs112698035	A	T	0.24	0.0158	0.0022	2.45E-12	51.57828328
ALM	rs112959543	G	A	0.6882	0.0154	0.002	2.93E-14	59.28973663
ALM	rs112963195	A	G	0.6049	-0.036	0.0019	1.05E-76	359.0011754
ALM	rs113063724	A	G	0.2362	-0.0221	0.0022	1.8E-23	100.9107088
ALM	rs113110183	T	G	0.3283	0.0224	0.002	1.76E-28	125.4394428
ALM	rs113222386	G	A	0.4753	-0.0142	0.0019	4.03E-14	55.85570756
ALM	rs113247306	A	G	0.4165	-0.0159	0.0019	1.36E-16	70.03015984
ALM	rs113540862	A	C	0.2224	-0.0156	0.0023	5.98E-12	46.00357637
ALM	rs113588432	G	A	0.1953	0.0131	0.0024	2.79E-08	29.79327043
ALM	rs113624671	C	G	0.3157	0.0309	0.002	6.89E-52	238.7014397
ALM	rs113870555	T	C	0.2444	-0.0172	0.0022	4.48E-15	61.12369543
ALM	rs113905685	C	T	0.5	0.0202	0.0019	1.4E-26	113.0299688
ALM	rs114085432	G	A	0.4995	-0.0157	0.0019	7.88E-17	68.27947509

ALM	rs114126607	G	A	0.4287	-0.0213	0.0019	2.05E-29	125.675342
ALM	rs114400038	C	T	0.356	-0.0137	0.002	4.29E-12	46.92229157
ALM	rs114998307	T	C	0.803	-0.021	0.0024	4.82E-19	76.56215991
ALM	rs115086545	T	C	0.158	-0.0301	0.0026	2.52E-31	134.0245526
ALM	rs115275418	T	G	0.0529	0.0321	0.0042	1.66E-14	58.41300583
ALM	rs115373889	G	A	0.4439	0.0232	0.0019	1.22E-33	149.0962906
ALM	rs115453753	A	C	0.7675	-0.0145	0.0022	6.97E-11	43.43988968
ALM	rs115467303	T	C	0.174	-0.0177	0.0025	1.02E-12	50.12617734
ALM	rs115557693	G	T	0.1426	-0.0189	0.0027	3.47E-12	48.99978234
ALM	rs115603745	T	C	0.4804	-0.0143	0.0019	2.05E-14	56.64517774
ALM	rs115647390	T	C	0.5051	-0.0295	0.0019	2.46E-55	241.0654112
ALM	rs115661045	C	T	0.036	0.0296	0.005	4.06E-09	35.04624432
ALM	rs115668337	G	T	0.1967	-0.0131	0.0024	2.61E-08	29.79327043
ALM	rs115690727	C	T	0.6905	-0.0145	0.002	8.91E-13	52.56226651
ALM	rs115806507	G	A	0.036	-0.0294	0.0051	6.36E-09	33.23168629
ALM	rs115825458	T	G	0.2836	-0.0185	0.0021	8.97E-19	77.60736501
ALM	rs115905998	G	T	0.3652	-0.015	0.002	1.33E-14	56.24975013
ALM	rs11593022	C	G	0.7299	0.0119	0.0021	2.34E-08	32.11096847
ALM	rs11599326	C	T	0.69	-0.0207	0.0021	6.85E-24	97.1628337
ALM	rs11599478	G	A	0.7297	-0.0171	0.0021	8.55E-16	66.30582791
ALM	rs116096645	A	G	0.4677	-0.0115	0.0019	1.04E-09	36.6341863
ALM	rs11619787	T	C	0.1381	-0.0162	0.0028	4.08E-09	33.4743411
ALM	rs11636217	T	C	0.3739	0.0141	0.002	5.41E-13	49.70227922
ALM	rs116365745	G	A	0.1703	-0.0164	0.0025	6.66E-11	43.03340884
ALM	rs116377033	C	T	0.246	-0.0145	0.0022	3.61E-11	43.43988968
ALM	rs116382426	C	T	0.1314	0.0226	0.0028	1.12E-15	65.14766979
ALM	rs116402126	G	A	0.336	0.0134	0.002	1.39E-11	44.8898006
ALM	rs116527002	A	G	0.1275	0.0193	0.0028	5.74E-12	47.51126854
ALM	rs11662742	C	T	0.6103	-0.0185	0.002	2.69E-21	85.56211993
ALM	rs11664595	G	A	0.0645	-0.0304	0.0039	4.02E-15	60.7597564
ALM	rs11665534	G	C	0.2856	-0.0297	0.0022	4.24E-42	182.2491904
ALM	rs116716197	G	A	0.2984	-0.0347	0.0021	1.61E-63	273.0350683
ALM	rs116765657	G	A	0.2436	-0.0181	0.0022	1.63E-16	67.68771586
ALM	rs116842830	A	G	0.0124	0.0536	0.0086	3.98E-10	38.84460841
ALM	rs116881537	T	C	0.4489	0.011	0.0019	1.07E-08	33.51785665
ALM	rs116898957	G	A	0.8375	-0.0184	0.0028	2.52E-11	43.18348165
ALM	rs116929779	C	G	0.3853	-0.0211	0.0019	1.08E-27	123.326322
ALM	rs11696257	C	T	0.8817	-0.0218	0.0029	1.13E-13	56.50866694
ALM	rs116963616	A	G	0.6139	0.0144	0.0019	1.36E-13	57.44018806
ALM	rs117014744	G	T	0.1784	-0.0419	0.0025	1.57E-64	280.8963522
ALM	rs11707828	G	A	0.1758	0.0164	0.0025	5.48E-11	43.03340884
ALM	rs117194100	C	T	0.4724	0.0109	0.0019	1.12E-08	32.91121115
ALM	rs117223734	A	G	0.3565	0.0126	0.002	1.38E-10	39.6898237
ALM	rs117225595	A	C	0.133	-0.0176	0.0028	2.58E-10	39.51002858
ALM	rs117239907	C	T	0.0971	-0.0277	0.0032	3.51E-18	74.93033122
ALM	rs117243209	C	T	0.0134	-0.0498	0.0082	1.18E-09	36.8832389
ALM	rs117288289	G	A	0.6717	0.0204	0.002	6.77E-24	104.0395378
ALM	rs117345726	G	T	0.1192	-0.0169	0.0029	8.3E-09	33.96061014
ALM	rs117358284	A	C	0.2043	0.0273	0.0024	3.38E-31	129.3900502

ALM	rs11735934	G	T	0.036	-0.0294	0.0051	6.35E-09	33.23168629
ALM	rs117388164	T	C	0.4696	-0.0169	0.0019	4.52E-19	79.11599205
ALM	rs11743718	C	T	0.2354	-0.0194	0.0022	1.89E-18	77.75998516
ALM	rs117439533	T	C	0.0286	-0.039	0.0056	4.83E-12	48.50106007
ALM	rs11749715	G	A	0.6397	-0.0189	0.002	5.64E-22	89.30210331
ALM	rs117511449	T	G	0.3165	-0.0193	0.002	2.66E-21	93.12208635
ALM	rs117534419	T	C	0.4101	-0.0238	0.0019	2.22E-35	156.9078903
ALM	rs117553175	A	C	0.4569	-0.0177	0.0019	1.03E-20	86.78354802
ALM	rs117608449	T	A	0.2724	-0.0166	0.0021	4.09E-15	62.48498321
ALM	rs117638558	A	G	0.2722	-0.0166	0.0021	3.93E-15	62.48498321
ALM	rs117707626	T	G	0.5415	0.017	0.0019	3.44E-19	80.05504605
ALM	rs117736998	A	G	0.3187	-0.0141	0.002	3.61E-12	49.70227922
ALM	rs117744655	T	C	0.127	0.0204	0.0028	7.98E-13	53.08139686
ALM	rs11775614	G	T	0.2337	-0.0204	0.0022	5.39E-20	85.98308913
ALM	rs117778582	C	T	0.4263	0.0154	0.0019	9.12E-16	65.69499904
ALM	rs11780275	C	T	0.6436	0.0135	0.002	7.05E-12	45.56229761
ALM	rs11789676	G	A	0.0561	-0.0342	0.0043	1.21E-15	63.25769629
ALM	rs117915876	G	A	0.1377	-0.0159	0.0027	7.43E-09	34.6788583
ALM	rs117933340	C	G	0.2544	-0.0321	0.0022	3.56E-49	212.8936824
ALM	rs117941643	T	C	0.3429	-0.0117	0.002	4.3E-09	34.22234798
ALM	rs117945327	C	T	0.3506	-0.0131	0.002	3.63E-11	42.90230943
ALM	rs117960958	G	A	0.0681	-0.0263	0.0038	2.68E-12	47.90075675
ALM	rs117982856	G	A	0.0774	-0.045	0.0036	4.92E-36	156.2493059
ALM	rs118110762	C	T	0.7331	-0.0121	0.0021	0.000000016	33.19939901
ALM	rs118166863	T	A	0.1731	-0.0158	0.0025	2.26E-10	39.94222257
ALM	rs118174104	G	A	0.1305	-0.0177	0.0028	2.98E-10	39.96028168
ALM	rs118188617	C	T	0.1135	-0.0201	0.003	1.97E-11	44.8898006
ALM	rs11869392	G	T	0.074	-0.022	0.0037	1.61E-09	35.35411615
ALM	rs11900472	T	G	0.747	-0.0135	0.0022	3.37E-10	37.65479141
ALM	rs12088743	T	C	0.5882	0.0174	0.0019	5.5E-20	83.86666347
ALM	rs12130472	A	C	0.0664	0.0216	0.0038	9.9E-09	32.31010578
ALM	rs12144911	T	C	0.8278	0.0185	0.0025	9.61E-14	54.75975675
ALM	rs12147388	T	C	0.7821	-0.0194	0.0023	3.75E-17	71.14524162
ALM	rs12168861	C	T	0.623	-0.0128	0.002	6.89E-11	40.95981805
ALM	rs12246741	G	A	0.0856	-0.0199	0.0034	3.84E-09	34.25676824
ALM	rs12325720	T	C	0.3578	0.025	0.002	6.28E-37	156.2493059
ALM	rs12356940	C	G	0.2295	-0.0126	0.0023	2.34E-08	30.01120884
ALM	rs12448741	T	G	0.1971	-0.0148	0.0024	6.15E-10	38.02760886
ALM	rs12499251	C	T	0.5572	-0.0184	0.0019	3.77E-22	93.78351693
ALM	rs12525884	A	C	0.2859	-0.0238	0.0021	3.41E-30	128.4438739
ALM	rs12533413	T	C	0.6026	-0.0274	0.0019	2.17E-45	207.9658352
ALM	rs12540834	T	C	0.2999	-0.0354	0.0021	5.37E-66	284.162003
ALM	rs12572369	G	A	0.5291	-0.0192	0.0019	5.65E-24	102.1158899
ALM	rs12591372	T	C	0.2507	0.017	0.0022	7.61E-15	59.71047856
ALM	rs12602643	T	A	0.6385	-0.0252	0.002	1.78E-37	158.7592948
ALM	rs12615915	C	T	0.2605	0.0243	0.0021	7.63E-30	133.8973644
ALM	rs12623288	G	A	0.1842	0.0285	0.0024	9.01E-32	141.0149986
ALM	rs12627890	C	T	0.5543	-0.017	0.0019	7.41E-19	80.05504605
ALM	rs12629445	A	G	0.4161	0.013	0.0019	1.1E-11	46.81419648

ALM	rs12659644	C	T	0.6896	-0.022	0.002	3.74E-27	120.9994625
ALM	rs12669720	A	G	0.2849	0.0198	0.0021	3.24E-21	88.89756429
ALM	rs12673776	G	A	0.1189	0.0185	0.0029	2.35E-10	40.6954197
ALM	rs12935933	T	G	0.4481	-0.0115	0.0019	1.59E-09	36.6341863
ALM	rs12952054	A	G	0.7438	-0.015	0.0022	3.8E-12	46.48739681
ALM	rs13006578	A	G	0.6458	0.0236	0.002	1.51E-33	139.2393815
ALM	rs13147761	G	A	0.1794	0.0147	0.0025	2.42E-09	34.57424642
ALM	rs13150933	T	C	0.6517	0.0134	0.002	1.36E-11	44.8898006
ALM	rs13155229	A	G	0.3727	-0.0232	0.0019	1.01E-32	149.0962906
ALM	rs13156286	G	A	0.246	0.0193	0.0023	1.46E-17	70.41367588
ALM	rs13188104	A	C	0.6554	-0.0119	0.002	1.99E-09	35.40234274
ALM	rs13353394	T	C	0.3596	0.0138	0.002	1.78E-12	47.60978851
ALM	rs1367697	G	T	0.0233	0.0411	0.0063	5.47E-11	42.55990165
ALM	rs138013293	C	A	0.4012	-0.0146	0.0019	4.02E-14	59.04682912
ALM	rs138088114	G	C	0.6509	0.0133	0.002	2.32E-11	44.22230356
ALM	rs138325129	G	A	0.5573	-0.0182	0.0019	7.9E-22	91.7558251
ALM	rs138370679	T	G	0.0681	-0.0262	0.0038	2.81E-12	47.53718496
ALM	rs138591337	C	T	0.3766	-0.028	0.0019	1.31E-47	217.1735505
ALM	rs139216405	C	G	0.3871	-0.0162	0.0019	5.81E-17	72.69773801
ALM	rs139376456	T	C	0.2026	-0.0147	0.0024	5.09E-10	37.51545835
ALM	rs139598183	T	A	0.2516	-0.0148	0.0022	9.85E-12	45.25599732
ALM	rs139809010	C	A	0.5684	-0.0108	0.0019	1.26E-08	32.31010578
ALM	rs139813858	G	A	0.3754	0.015	0.0022	9.74E-12	46.48739681
ALM	rs139825196	T	G	0.335	-0.0205	0.002	8.39E-25	105.0620333
ALM	rs140238097	G	A	0.2634	-0.0127	0.0021	3.93E-09	36.57353368
ALM	rs1402951	G	A	0.1571	-0.0551	0.0026	2.07E-100	449.1119103
ALM	rs140385166	T	C	0.2355	0.0131	0.0022	4.16E-09	35.45645407
ALM	rs140506908	C	A	0.4399	-0.0188	0.0019	3.88E-23	97.90538227
ALM	rs140577562	A	C	0.6681	0.0431	0.002	2.63E-104	464.4004371
ALM	rs140829999	C	G	0.2845	0.0167	0.0021	1.13E-15	63.2400819
ALM	rs140942363	G	A	0.1837	0.0335	0.0025	3.39E-41	179.5592024
ALM	rs140943336	C	T	0.768	-0.0162	0.0022	6.26E-13	54.22289963
ALM	rs141100874	C	T	0.5859	0.0169	0.0019	2.06E-18	79.11599205
ALM	rs141200065	A	G	0.5622	0.0164	0.0019	1.01E-17	74.50382417
ALM	rs141204412	G	A	0.3249	-0.0434	0.002	1.21E-103	470.8879083
ALM	rs141282824	G	A	0.7333	-0.0199	0.0021	1.42E-20	89.79778705
ALM	rs141399292	G	A	0.7104	-0.0141	0.0021	1.56E-11	45.0814324
ALM	rs141419457	T	C	0.6909	-0.0144	0.002	1.08E-12	51.83976972
ALM	rs141512479	A	G	0.5369	0.018	0.0019	1.26E-21	89.75029384
ALM	rs141918496	A	C	0.336	0.0134	0.002	1.69E-11	44.8898006
ALM	rs142031671	A	G	0.3146	-0.0117	0.0021	1.23E-08	31.04067844
ALM	rs142035563	T	A	0.0311	-0.0385	0.0054	1.24E-12	50.83139286
ALM	rs142685384	G	A	0.7578	0.0175	0.0022	2.58E-15	63.27451232
ALM	rs142807707	A	C	0.3058	-0.0119	0.002	5.9E-09	35.40234274
ALM	rs143067350	A	G	0.6982	-0.0209	0.0021	6.8E-24	99.04944664
ALM	rs143444153	G	A	0.7427	-0.0442	0.0022	3.39E-93	403.6428351
ALM	rs143621475	C	T	0.275	-0.0118	0.0021	2.27E-08	31.57355589
ALM	rs143651461	G	A	0.1028	0.0314	0.0031	5.46E-24	102.5968387
ALM	rs143663020	C	A	0.7248	-0.0166	0.0021	8.16E-15	62.48498321

ALM	rs144433223	A	G	0.4759	-0.0128	0.0019	1.7E-11	45.38483995
ALM	rs144509180	A	C	0.4903	0.0154	0.0019	3.89E-16	65.69499904
ALM	rs144687466	C	T	0.321	0.0217	0.002	3.05E-27	117.7219771
ALM	rs145026158	G	A	0.6117	0.0278	0.0019	8.52E-47	214.0821515
ALM	rs145421042	A	T	0.3377	0.0215	0.002	8.44E-27	115.5619867
ALM	rs145483500	G	A	0.6923	-0.016	0.0021	5.76E-15	58.04962876
ALM	rs145686897	C	T	0.1865	-0.0136	0.0024	2.81E-08	32.11096847
ALM	rs145788018	G	T	0.6626	0.0159	0.002	2.19E-15	63.20221925
ALM	rs146466019	G	A	0.5323	-0.0308	0.0019	1.09E-58	262.7799961
ALM	rs146547466	G	A	0.5774	0.0154	0.0019	1.09E-15	65.69499904
ALM	rs146684367	C	T	0.1847	-0.0303	0.0025	7.13E-35	146.8937475
ALM	rs146721402	G	A	0.385	-0.0231	0.0019	2.73E-33	147.8137478
ALM	rs146901724	G	A	0.2639	-0.0235	0.0021	4.29E-28	125.2262011
ALM	rs146972540	C	T	0.4181	-0.0173	0.0019	6.45E-20	82.9054489
ALM	rs147237469	G	A	0.4137	-0.0196	0.002	5.15E-23	96.03957339
ALM	rs147573701	T	C	0.9305	-0.0333	0.0038	1.29E-18	76.79259517
ALM	rs147609952	G	A	0.4021	0.0224	0.0019	1.98E-31	138.9910723
ALM	rs147610882	C	T	0.8408	0.0231	0.0027	3.6E-17	73.19720572
ALM	rs147795073	A	G	0.1025	-0.0356	0.0031	5.42E-30	131.8787066
ALM	rs147804259	G	A	0.3505	-0.0133	0.002	2.08E-11	44.22230356
ALM	rs147982856	A	G	0.9188	-0.0298	0.0035	9.21E-18	72.49273921
ALM	rs148077221	C	A	0.1873	0.04	0.0024	1.41E-60	277.7765439
ALM	rs148288528	A	G	0.1935	0.0305	0.0024	8.43E-38	161.5010187
ALM	rs148351327	T	C	0.1796	-0.0244	0.0025	3.52E-23	95.25717686
ALM	rs148506766	C	T	0.4902	-0.0152	0.0019	1.03E-15	63.99971571
ALM	rs148624001	G	A	0.1352	0.0249	0.0028	1.73E-19	79.08255687
ALM	rs148767773	C	T	0.2565	-0.018	0.0021	5.39E-17	73.4690614
ALM	rs148769612	T	C	0.1733	-0.0178	0.0025	8.12E-13	50.69417481
ALM	rs148906623	A	G	0.5627	0.0291	0.0019	1.99E-52	234.5723652
ALM	rs148938637	C	T	0.4871	0.0136	0.0019	7.66E-13	51.23522947
ALM	rs149202743	A	T	0.165	-0.0179	0.0026	3.88E-12	47.39771845
ALM	rs149254829	C	T	0.3391	-0.0163	0.002	1.86E-16	66.42220495
ALM	rs149495345	T	C	0.1839	0.0147	0.0024	1.39E-09	37.51545835
ALM	rs149660134	T	G	0.0115	-0.0602	0.0096	3.78E-10	39.32317602
ALM	rs149900441	C	T	0.9476	0.0274	0.0042	9.39E-11	42.55990165
ALM	rs150315805	T	C	0.3519	0.0157	0.002	4.2E-15	61.62222627
ALM	rs150425738	G	A	0.5426	0.0159	0.0019	3.09E-17	70.03015984
ALM	rs150619316	T	A	0.0525	-0.0246	0.0042	5.65E-09	34.30597006
ALM	rs150637397	A	G	0.6903	-0.0206	0.0021	1.02E-23	96.22632993
ALM	rs150735542	A	C	0.4866	0.015	0.0019	1.99E-15	62.32659295
ALM	rs150885624	G	C	0.1532	0.0252	0.0027	3.33E-20	87.11072416
ALM	rs151054415	C	T	0.026	-0.0401	0.0069	5.33E-09	33.77447715
ALM	rs151121248	T	C	0.2503	-0.0192	0.0022	1.54E-18	76.16495093
ALM	rs151167959	C	T	0.4757	-0.0141	0.0019	5.78E-14	55.07177753
ALM	rs151323928	G	A	0.0432	-0.0258	0.0046	2.69E-08	31.45732718
ALM	rs1542899	A	G	0.165	0.02	0.0026	5.18E-15	59.17133479
ALM	rs1590454	T	A	0.2527	-0.0521	0.0022	7.67E-126	560.8260212
ALM	rs159523	C	T	0.7554	-0.0143	0.0022	6.2E-11	42.24981232
ALM	rs1603201	T	G	0.6445	-0.0126	0.002	1.75E-10	39.6898237

ALM	rs1608598	A	T	0.6814	0.0134	0.002	3.31E-11	44.8898006
ALM	rs1668596	A	G	0.2566	0.0145	0.0022	2.34E-11	43.43988968
ALM	rs1675791	G	A	0.6962	0.0331	0.002	1.39E-58	273.9012833
ALM	rs16827109	T	G	0.1755	-0.0142	0.0025	1.14E-08	32.26225669
ALM	rs16844312	T	C	0.4728	-0.0143	0.0019	1.97E-14	56.64517774
ALM	rs16952817	C	T	0.6512	0.0133	0.002	1.88E-11	44.22230356
ALM	rs17048976	G	A	0.318	0.0215	0.002	1.13E-26	115.5619867
ALM	rs17084870	G	T	0.6881	0.0155	0.002	2.95E-14	60.0622332
ALM	rs17093824	A	G	0.1491	-0.0159	0.0027	2.23E-09	34.6788583
ALM	rs17135258	G	A	0.116	-0.0346	0.003	9.49E-32	133.0171869
ALM	rs17136392	G	A	0.2622	-0.0279	0.0023	1.66E-34	147.1467944
ALM	rs17149981	T	C	0.6242	-0.0113	0.002	6.37E-09	31.9223582
ALM	rs17198173	C	G	0.1016	0.0215	0.0031	6.06E-12	48.10072286
ALM	rs17235284	G	A	0.3573	-0.0218	0.002	1.5E-28	118.8094722
ALM	rs17308085	T	C	0.3559	0.0159	0.002	9.78E-16	63.20221925
ALM	rs17358216	C	T	0.1441	0.0185	0.0027	5.07E-12	46.94766526
ALM	rs17373408	C	T	0.1313	-0.0221	0.0029	1.58E-14	58.07465285
ALM	rs17388106	T	C	0.2855	-0.0176	0.0021	6.33E-17	70.2400508
ALM	rs17450969	G	A	0.2467	0.0281	0.0022	2.35E-37	163.1418373
ALM	rs17500863	G	A	0.0789	-0.0194	0.0035	0.000000034	30.72312883
ALM	rs17505447	C	T	0.2064	-0.0306	0.0023	1.42E-39	177.0048848
ALM	rs17666791	T	C	0.7533	0.0124	0.0022	1.39E-08	31.76845392
ALM	rs17691788	G	A	0.2027	0.0148	0.0024	3.1E-10	38.02760886
ALM	rs17736491	G	A	0.5425	0.0142	0.0019	7.13E-14	55.85570756
ALM	rs17737019	G	A	0.283	0.0171	0.0021	3.79E-16	66.30582791
ALM	rs17745069	A	G	0.1867	0.0206	0.0024	2.06E-17	73.67328385
ALM	rs17879478	T	G	0.3531	0.0174	0.002	7.07E-19	75.68966378
ALM	rs17882852	A	G	0.19	-0.0209	0.0024	6.15E-18	75.83473258
ALM	rs180763827	C	T	0.3603	0.0131	0.002	1.34E-10	42.90230943
ALM	rs181705334	C	T	0.6978	0.0212	0.0021	6.52E-25	101.9133795
ALM	rs181930270	C	T	0.3598	0.0137	0.002	2.44E-12	46.92229157
ALM	rs182254564	T	C	0.0298	-0.036	0.0055	6.27E-11	42.8427849
ALM	rs182565136	T	C	0.4281	-0.011	0.0019	9.22E-09	33.51785665
ALM	rs183020856	T	G	0.4696	-0.0169	0.0019	4.4E-19	79.11599205
ALM	rs183119238	C	G	0.0432	-0.026	0.0046	2.09E-08	31.94692803
ALM	rs183158901	A	C	0.1814	0.0291	0.0024	5.6E-33	147.0149719
ALM	rs183194519	A	G	0.1195	0.0175	0.0029	2.02E-09	36.41482041
ALM	rs183409197	A	C	0.5734	0.0287	0.0019	3.47E-51	228.1679615
ALM	rs184692991	G	A	0.2613	-0.0228	0.0022	5.26E-26	107.4044816
ALM	rs185746828	C	T	0.4201	-0.015	0.0019	5.38E-15	62.32659295
ALM	rs186178365	C	T	0.3707	-0.0151	0.0022	1.5E-11	47.10929487
ALM	rs186358560	G	C	0.3541	-0.0197	0.002	2.17E-23	97.02206902
ALM	rs186405483	G	C	0.6167	0.0191	0.0019	7.66E-23	101.0549528
ALM	rs186969651	C	T	0.3825	0.0148	0.0019	3.24E-14	60.67563075
ALM	rs1870623	G	A	0.3657	0.0197	0.002	3.29E-23	97.02206902
ALM	rs1874332	C	T	0.1896	0.014	0.0024	6.48E-09	34.02762662
ALM	rs187599638	C	T	0.4288	-0.0282	0.0019	1.14E-48	220.2871101
ALM	rs188445124	A	G	0.5622	0.0122	0.0019	1.39E-10	41.22973375
ALM	rs188609351	T	A	0.3272	0.0225	0.002	7.8E-29	126.5619378

ALM	rs190712058	T	C	0.5452	0.0295	0.0019	5.2E-54	241.0654112
ALM	rs190832171	G	C	0.3296	0.011	0.002	4.86E-08	30.24986563
ALM	rs191857033	T	C	0.0335	0.0323	0.0053	1.03E-09	37.14081045
ALM	rs191908519	G	A	0.235	0.0177	0.0022	2.5E-15	64.72905131
ALM	rs1920390	C	A	0.7134	0.0243	0.0021	2.55E-31	133.8973644
ALM	rs192348238	T	G	0.1597	0.0341	0.0026	3.02E-40	172.0125495
ALM	rs192753507	G	A	0.7879	0.016	0.0023	7.22E-12	48.39297974
ALM	rs193229035	A	G	0.5077	-0.011	0.0019	4.33E-09	33.51785665
ALM	rs2038789	A	G	0.2139	-0.0614	0.0023	9.91E-155	712.6546793
ALM	rs206224	G	A	0.6168	0.0107	0.002	4.48E-08	28.62237286
ALM	rs2082923	T	C	0.3379	0.0118	0.002	4.16E-09	34.80984537
ALM	rs2279417	G	C	0.1779	0.0164	0.0025	3.85E-11	43.03340884
ALM	rs2299930	C	T	0.0909	0.0199	0.0033	1.65E-09	36.3643931
ALM	rs231016	T	C	0.2391	-0.0125	0.0022	1.74E-08	32.28291445
ALM	rs2319080	A	G	0.4964	-0.0161	0.0019	2.27E-17	71.80300515
ALM	rs2453300	C	T	0.356	-0.0218	0.002	1.95E-28	118.8094722
ALM	rs2485346	G	T	0.4699	0.0205	0.0019	1.4E-27	116.4122253
ALM	rs2492286	G	T	0.7784	0.0274	0.0023	1.92E-33	141.9199745
ALM	rs2582734	C	A	0.3395	0.0129	0.002	7.31E-11	41.6023152
ALM	rs258555	T	C	0.1727	0.0214	0.0025	7.81E-18	73.27327452
ALM	rs2711103	G	T	0.2477	0.0178	0.0022	4.29E-16	65.46251913
ALM	rs2725802	T	C	0.2495	-0.0199	0.0022	1.51E-19	81.81988448
ALM	rs2808121	T	C	0.3588	0.0243	0.002	4.3E-35	147.6218443
ALM	rs28417297	A	G	0.2468	0.0175	0.0022	1.76E-15	63.27451232
ALM	rs2843438	C	T	0.6678	-0.0238	0.002	5.44E-32	141.609371
ALM	rs2850907	C	T	0.41	-0.0112	0.0019	5.98E-09	34.74776809
ALM	rs28550012	T	C	0.7352	0.0166	0.0021	7.87E-15	62.48498321
ALM	rs28665735	G	T	0.2614	-0.0228	0.0022	4.48E-26	107.4044816
ALM	rs28721287	G	T	0.5595	0.0171	0.0019	1.37E-19	80.99964019
ALM	rs2873895	T	C	0.557	-0.0138	0.0019	4.35E-13	52.75322827
ALM	rs2886982	G	A	0.3921	-0.0116	0.0019	1.76E-09	37.27407265
ALM	rs28874654	G	A	0.3586	0.0141	0.002	7.51E-13	49.70227922
ALM	rs28886995	T	C	0.3723	0.0142	0.002	3.9E-13	50.40977608
ALM	rs2955037	G	A	0.4605	0.0248	0.0019	9.33E-39	170.3704343
ALM	rs2968828	C	A	0.4378	-0.0128	0.0019	1.61E-11	45.38483995
ALM	rs2998005	A	G	0.2461	0.0166	0.0022	4.69E-14	56.93363139
ALM	rs3012721	G	A	0.1945	-0.0159	0.0025	1.68E-10	40.44942032
ALM	rs308421	A	G	0.3781	-0.0121	0.002	6.72E-10	36.60233741
ALM	rs3093860	A	G	0.3874	-0.0152	0.0019	3.02E-15	63.99971571
ALM	rs30985	T	C	0.0235	0.0418	0.0063	2.2E-11	44.02197628
ALM	rs317809	G	A	0.3439	0.0307	0.002	8.87E-52	235.6214534
ALM	rs317817	A	C	0.2326	0.0488	0.0022	1.18E-104	492.0308722
ALM	rs341092	T	C	0.7792	0.0172	0.0023	5.18E-14	55.92413721
ALM	rs34236778	C	A	0.601	-0.011	0.0019	1.03E-08	33.51785665
ALM	rs34311786	C	T	0.1133	-0.0288	0.003	5.35E-22	92.15959062
ALM	rs34495603	C	T	0.5164	0.0117	0.0019	4.74E-10	37.91949915
ALM	rs34499286	A	G	0.3795	-0.0122	0.0019	3.88E-10	41.22973375
ALM	rs34619208	T	C	0.341	-0.0174	0.002	2.15E-18	75.68966378
ALM	rs35046933	A	G	0.1852	0.0209	0.0024	6.95E-18	75.83473258

ALM	rs35329807	A	G	0.2024	0.0216	0.0023	3E-20	88.19620558
ALM	rs35491793	G	A	0.0891	0.0389	0.0033	2.63E-32	138.9534691
ALM	rs35843825	C	T	0.7415	-0.0199	0.0022	3.17E-20	81.81988448
ALM	rs35869274	T	C	0.2882	0.012	0.0021	1.26E-08	32.65291618
ALM	rs35966599	A	G	0.0596	0.0263	0.004	3.94E-11	43.23043297
ALM	rs36012757	T	C	0.2003	-0.0136	0.0023	6.46E-09	34.96392786
ALM	rs36103144	G	T	0.5527	-0.0112	0.0019	4.03E-09	34.74776809
ALM	rs36159865	G	A	0.1266	-0.0158	0.0028	0.000000028	31.84169529
ALM	rs374604791	G	A	0.574	-0.0168	0.0019	4.78E-18	78.18247819
ALM	rs375562699	C	T	0.4273	0.0321	0.0019	1.92E-63	285.4308651
ALM	rs3779065	C	T	0.0483	-0.0282	0.0044	2.08E-10	41.07626382
ALM	rs4011833	A	G	0.2754	0.0144	0.0022	5.3E-11	42.8427849
ALM	rs409803	A	G	0.1018	0.0286	0.0031	3.9E-20	85.1151266
ALM	rs4131643	T	C	0.1278	-0.0187	0.0028	4.66E-11	44.6031182
ALM	rs4267616	C	T	0.1882	0.015	0.0024	5.46E-10	39.06232648
ALM	rs4324319	C	T	0.5711	-0.0125	0.0019	3.55E-11	43.28235621
ALM	rs434026	G	T	0.1401	0.0412	0.0027	4.33E-52	232.8439588
ALM	rs4419434	G	A	0.3588	0.0111	0.002	1.99E-08	30.80236317
ALM	rs4449055	C	T	0.0218	-0.0753	0.0065	2.96E-31	134.2027175
ALM	rs4475224	A	G	0.6327	0.0182	0.002	1.31E-20	82.80963215
ALM	rs448142	T	C	0.5215	-0.0161	0.0019	2.12E-17	71.80300515
ALM	rs45456095	C	T	0.5537	-0.0144	0.0019	4.13E-14	57.44018806
ALM	rs45463891	T	G	0.5218	-0.0235	0.0019	1.46E-35	152.9771598
ALM	rs45598837	T	C	0.0489	-0.0288	0.0044	6.11E-11	42.8427849
ALM	rs4688295	T	C	0.7706	0.013	0.0022	6.65E-09	34.91720027
ALM	rs4698524	C	T	0.5927	0.0151	0.0019	4.45E-15	63.16038426
ALM	rs4741946	A	G	0.4551	0.0121	0.0019	1.85E-10	40.55660655
ALM	rs482639	A	C	0.4514	0.0285	0.0019	8.8E-52	224.9990005
ALM	rs4894509	G	A	0.2307	0.0266	0.0022	1.41E-32	146.1894333
ALM	rs4923880	G	A	0.0247	-0.0446	0.0061	3.95E-13	53.45743521
ALM	rs4925362	G	T	0.9181	-0.0205	0.0035	2.89E-09	34.30597006
ALM	rs493041	T	C	0.6045	0.018	0.0019	1.99E-20	89.75029384
ALM	rs519305	G	A	0.5938	-0.0189	0.0019	1.44E-22	98.94969896
ALM	rs536810938	G	A	0.7083	-0.0137	0.0021	4.15E-11	42.55990165
ALM	rs540256462	C	T	0.6221	0.015	0.0019	1.35E-14	62.32659295
ALM	rs543222455	T	C	0.7008	0.0321	0.0021	6.31E-55	233.6520233
ALM	rs544649621	A	G	0.7785	-0.0167	0.0023	2.29E-13	52.71999266
ALM	rs547121038	C	T	0.4316	0.0106	0.0019	3.36E-08	31.12451548
ALM	rs55634712	T	C	0.0645	-0.0304	0.0039	4.82E-15	60.7597564
ALM	rs55782859	C	G	0.1239	-0.0172	0.0029	2.83E-09	35.17701378
ALM	rs55784400	T	C	0.7554	-0.056	0.0022	4.59E-144	647.9310061
ALM	rs55814401	T	G	0.199	-0.0144	0.0024	1.44E-09	35.99984009
ALM	rs55850097	C	T	0.4174	0.0131	0.0019	7.39E-12	47.53718496
ALM	rs55991305	C	T	0.1128	-0.0203	0.003	7.24E-12	45.78757439
ALM	rs56048552	C	T	0.3881	-0.0164	0.0019	2.64E-17	74.50382417
ALM	rs56052346	C	T	0.0947	-0.0191	0.0033	4.33E-09	33.49939206
ALM	rs56117556	A	G	0.0197	-0.0807	0.007	1.46E-30	132.9073688
ALM	rs56186101	C	T	0.042	-0.0279	0.0047	2.88E-09	35.23796027
ALM	rs56191582	C	T	0.2487	-0.0392	0.0022	4.46E-73	317.486193

ALM	rs56197302	G	A	0.6042	-0.0165	0.0019	1.47E-17	75.41517747
ALM	rs56230562	A	T	0.3872	0.0126	0.0019	8.46E-11	43.97764398
ALM	rs56291080	G	A	0.1231	-0.0174	0.0029	1.53E-09	35.99984009
ALM	rs56306815	A	C	0.4903	-0.0245	0.0019	3.29E-39	166.2734996
ALM	rs56308818	T	C	0.1721	0.0281	0.0025	7.43E-30	126.3370388
ALM	rs56750563	C	G	0.243	0.0162	0.0022	2.88E-13	54.22289963
ALM	rs57331385	G	A	0.6097	0.0303	0.0019	2.99E-55	254.3174299
ALM	rs57359368	G	A	0.5853	-0.0153	0.0019	8.85E-16	64.8445873
ALM	rs57480975	T	G	0.68	-0.0166	0.002	2.37E-16	68.88969399
ALM	rs57650377	C	T	0.3181	0.0198	0.002	1.93E-22	98.00956464
ALM	rs58067236	A	G	0.612	-0.0132	0.0019	7.06E-12	48.26571358
ALM	rs581884	A	G	0.157	-0.0552	0.0026	1.19E-100	450.7435599
ALM	rs58759531	C	T	0.57	-0.0109	0.0019	8.76E-09	32.91121115
ALM	rs59494350	C	T	0.5382	-0.0193	0.0019	1.24E-24	103.1823671
ALM	rs59723054	A	T	0.7566	0.0131	0.0022	2.75E-09	35.45645407
ALM	rs5994254	G	A	0.1973	-0.0212	0.0024	1.55E-18	78.02743117
ALM	rs60196381	T	C	0.6243	-0.0123	0.0019	2.41E-10	41.9084011
ALM	rs60513865	C	T	0.6209	-0.0185	0.002	5.49E-21	85.56211993
ALM	rs60537431	C	A	0.0706	0.0357	0.0037	1.77E-22	93.09600721
ALM	rs6061090	C	T	0.174	0.0581	0.0025	4.47E-119	540.0952009
ALM	rs60941356	C	T	0.4575	-0.0205	0.0019	8.55E-27	116.4122253
ALM	rs61141120	C	T	0.0958	-0.0328	0.0032	4.73E-24	105.0620333
ALM	rs6117853	G	A	0.4177	-0.0117	0.0019	9.74E-10	37.91949915
ALM	rs61591274	G	T	0.5429	0.0118	0.0019	5.34E-10	38.57046579
ALM	rs61766734	T	A	0.2713	0.012	0.0021	1.37E-08	32.65291618
ALM	rs61773028	T	C	0.1378	-0.0155	0.0027	1.56E-08	32.95595786
ALM	rs61822873	C	T	0.6288	-0.0157	0.002	8.55E-16	61.62222627
ALM	rs61862290	C	G	0.1748	0.0288	0.0025	1.06E-30	132.7098105
ALM	rs61941622	C	T	0.6995	0.015	0.0021	2.71E-13	51.02018153
ALM	rs62033249	G	A	0.1331	-0.0176	0.0028	2.65E-10	39.51002858
ALM	rs62056331	G	C	0.1809	-0.0387	0.0025	5.8E-56	239.6293356
ALM	rs62061499	A	G	0.5257	-0.0104	0.0019	4.33E-08	29.96108575
ALM	rs62095712	A	G	0.0684	-0.0266	0.0038	1.49E-12	48.99978234
ALM	rs62173768	T	C	0.5585	-0.0105	0.0019	2.39E-08	30.54003054
ALM	rs62270783	C	T	0.7787	0.0246	0.0023	1.81E-27	114.3964673
ALM	rs62275710	C	T	0.4307	-0.0143	0.0019	7.14E-14	56.64517774
ALM	rs62278682	G	A	0.4159	0.013	0.0019	1.15E-11	46.81419648
ALM	rs62321864	G	C	0.7765	-0.0255	0.0023	3.52E-28	122.9200589
ALM	rs62344530	C	T	0.4357	-0.0158	0.0019	1.4E-16	69.15204739
ALM	rs62361810	G	C	0.8573	-0.0195	0.0027	4.33E-13	52.16026213
ALM	rs62432531	G	A	0.2444	-0.0175	0.0022	2E-15	63.27451232
ALM	rs62460527	C	T	0.0616	-0.0411	0.0039	1.83E-25	111.0586783
ALM	rs62513975	G	A	0.4192	-0.0268	0.0019	2.76E-44	198.957565
ALM	rs62519875	C	G	0.0564	0.0268	0.0041	9.75E-11	42.72675845
ALM	rs640466	T	C	0.5236	-0.0163	0.0019	1.03E-17	73.59801102
ALM	rs6494452	G	A	0.7232	-0.028	0.0021	1.21E-39	177.7769881
ALM	rs6544501	G	A	0.13	-0.0238	0.0028	1.54E-17	72.24967906
ALM	rs655334	A	T	0.7144	-0.0175	0.0021	3.65E-17	69.44413597
ALM	rs6557547	T	C	0.5597	0.0236	0.0019	2.66E-35	154.2818631

ALM	rs662776	G	T	0.2336	-0.0204	0.0022	6.13E-20	85.98308913
ALM	rs66578509	A	G	0.2089	-0.0144	0.0023	7.72E-10	39.19831359
ALM	rs6670799	G	A	0.1979	0.0301	0.0024	1.22E-37	157.2927041
ALM	rs6700043	T	G	0.2619	0.0398	0.0021	1.02E-76	359.1911482
ALM	rs6726675	A	C	0.6177	0.0148	0.0019	1.99E-14	60.67563075
ALM	rs6729220	G	A	0.8582	0.0158	0.0027	4.45E-09	34.24401798
ALM	rs67300624	T	G	0.2574	0.0132	0.0021	7.77E-10	39.51002858
ALM	rs6734041	G	A	0.3264	0.0127	0.002	1.9E-10	40.32232089
ALM	rs6769757	C	T	0.2227	-0.016	0.0023	1.57E-12	48.39297974
ALM	rs684417	C	T	0.4453	0.0108	0.0019	1.63E-08	32.31010578
ALM	rs6905364	C	T	0.4985	0.0139	0.0019	1.66E-13	53.52053788
ALM	rs700124	G	A	0.429	-0.0119	0.0019	5.06E-10	39.22697257
ALM	rs700125	G	A	0.4475	-0.0138	0.0019	4E-13	52.75322827
ALM	rs7010827	A	G	0.1428	-0.0167	0.0027	6.5E-10	38.25634584
ALM	rs7031105	T	C	0.7968	0.0135	0.0024	1.45E-08	31.64048445
ALM	rs7087849	T	G	0.2896	-0.0144	0.0021	6.33E-12	47.0201993
ALM	rs7128537	G	A	0.2757	-0.0268	0.0021	6.84E-37	162.8654897
ALM	rs7139145	C	A	0.2048	0.0301	0.0023	5.97E-38	171.2676702
ALM	rs7146716	T	C	0.2918	0.0274	0.0021	2.4E-39	170.2396066
ALM	rs71529207	C	T	0.2857	0.0331	0.0021	3.86E-56	248.4365382
ALM	rs7156684	C	T	0.6957	-0.0351	0.0021	7.23E-65	279.366106
ALM	rs7166638	G	C	0.3472	-0.0198	0.002	2.4E-22	98.00956464
ALM	rs7175541	A	C	0.4135	-0.0141	0.0019	2.86E-13	55.07177753
ALM	rs72663780	T	C	0.2202	0.0327	0.0023	2.13E-47	202.1333176
ALM	rs72688441	G	A	0.3026	0.0137	0.002	1.52E-11	46.92229157
ALM	rs72696873	T	G	0.5768	-0.0192	0.0019	4.69E-24	102.1158899
ALM	rs72713061	T	A	0.9013	-0.0346	0.0032	1.66E-27	116.9096369
ALM	rs72727410	G	A	0.3941	0.0159	0.0019	1.85E-16	70.03015984
ALM	rs72755948	T	C	0.6689	0.0164	0.002	2.16E-16	67.23970132
ALM	rs72759135	G	A	0.2489	0.0253	0.0022	5.37E-30	132.2494125
ALM	rs72763065	C	T	0.3734	0.0139	0.002	1.21E-12	48.30228544
ALM	rs72780205	G	A	0.248	0.0589	0.0022	9.66E-160	716.7757417
ALM	rs72783723	C	T	0.3875	-0.0159	0.0019	1.75E-16	70.03015984
ALM	rs72813287	T	G	0.3092	-0.0112	0.002	3.78E-08	31.3598607
ALM	rs72829896	G	A	0.1183	-0.0187	0.0029	1.74E-10	41.58007689
ALM	rs72841383	T	C	0.3796	-0.0119	0.002	1.17E-09	35.40234274
ALM	rs72867279	C	A	0.402	0.0224	0.0019	2.6E-31	138.9910723
ALM	rs72892458	A	G	0.457	-0.0177	0.0019	9.8E-21	86.78354802
ALM	rs72900830	C	T	0.6909	-0.0145	0.002	8.3E-13	52.56226651
ALM	rs72906665	A	G	0.036	0.0296	0.005	4.09E-09	35.04624432
ALM	rs72935795	A	T	0.6266	0.0178	0.0019	6.54E-20	87.76692315
ALM	rs72993506	T	A	0.6646	-0.0246	0.002	1.85E-34	151.289328
ALM	rs72993555	T	C	0.5723	-0.0171	0.0019	5.65E-19	80.99964019
ALM	rs73036649	T	C	0.0625	0.056	0.0039	5.51E-47	206.1792288
ALM	rs73147447	T	C	0.1531	0.0248	0.0026	5.28E-21	90.98184437
ALM	rs73233329	G	T	0.0204	0.0743	0.0069	3.16E-27	115.9518059
ALM	rs7324667	G	A	0.2311	-0.0167	0.0023	1.1E-13	52.71999266
ALM	rs733379	G	A	0.7067	0.0133	0.0021	1.66E-10	40.11093294
ALM	rs73361256	T	A	0.3068	0.0169	0.0021	1.86E-16	64.76388465

ALM	rs73466917	T	C	0.7429	-0.0139	0.0022	1.51E-10	39.91924416
ALM	rs73583779	A	G	0.2755	0.0116	0.0021	4.29E-08	30.51233612
ALM	rs73722924	G	A	0.5097	0.0151	0.0019	1.75E-15	63.16038426
ALM	rs73813008	T	A	0.1176	-0.0375	0.0029	1.68E-37	167.21091
ALM	rs74316133	G	A	0.4339	0.0126	0.0019	3.51E-11	43.97764398
ALM	rs74371975	T	C	0.4245	-0.0275	0.0019	6.07E-47	209.4866041
ALM	rs74381675	C	T	0.1053	-0.0169	0.0031	4.17E-08	29.71995123
ALM	rs74947462	A	G	0.461	0.0132	0.0019	2.91E-12	48.26571358
ALM	rs75062476	A	C	0.0655	-0.0294	0.0038	1.46E-14	59.85845987
ALM	rs75069926	G	A	0.4893	-0.0106	0.0019	1.94E-08	31.12451548
ALM	rs75077790	C	T	0.5256	0.0183	0.0019	5.42E-22	92.76690094
ALM	rs7519946	A	G	0.3702	0.0141	0.0019	4.68E-13	55.07177753
ALM	rs75217126	T	A	0.4166	-0.0114	0.0019	2.91E-09	35.99984009
ALM	rs7534418	T	C	0.1784	-0.0139	0.0024	1.23E-08	33.54325378
ALM	rs75349691	A	G	0.7	-0.0216	0.0021	1.31E-25	105.7954484
ALM	rs75487350	G	A	0.3242	0.0147	0.002	3.63E-13	54.02226003
ALM	rs75742758	C	T	0.599	-0.0118	0.002	1.99E-09	34.80984537
ALM	rs7589696	C	T	0.7201	-0.0118	0.0021	1.56E-08	31.57355589
ALM	rs7591141	C	T	0.1304	-0.0239	0.0028	1.42E-17	72.85809473
ALM	rs75929899	G	A	0.1391	-0.0166	0.0027	1.16E-09	37.79955774
ALM	rs7597223	C	T	0.5493	0.0282	0.0019	9.5E-51	220.2871101
ALM	rs76001666	C	T	0.5526	0.0173	0.0019	1.59E-19	82.9054489
ALM	rs76048092	T	A	0.6663	-0.0114	0.002	1.25E-08	32.48985568
ALM	rs7604842	C	T	0.324	-0.0111	0.002	3.13E-08	30.80236317
ALM	rs7605241	T	C	0.2026	0.0247	0.0023	6.16E-26	115.3284102
ALM	rs7609845	A	G	0.2105	-0.0183	0.0023	2.07E-15	63.30595698
ALM	rs76124421	T	C	0.5768	0.0132	0.0019	5.46E-12	48.26571358
ALM	rs76467480	A	G	0.1362	0.0261	0.0027	2.06E-21	93.44402936
ALM	rs76562100	C	A	0.6058	-0.0587	0.0019	1.59E-200	954.4805247
ALM	rs76676382	A	G	0.4177	-0.0118	0.0019	8.99E-10	38.57046579
ALM	rs76698819	G	T	0.3511	-0.0146	0.002	8.64E-14	53.28976328
ALM	rs76728732	T	C	0.245	-0.0121	0.0022	4.48E-08	30.24986563
ALM	rs76740160	T	C	0.1308	-0.0239	0.0029	1.25E-16	67.92003123
ALM	rs76890996	G	A	0.5154	-0.0111	0.0019	4.37E-09	34.1300423
ALM	rs76905997	C	T	0.2503	-0.0154	0.0022	2.59E-12	48.99978234
ALM	rs76916044	G	A	0.3982	0.0229	0.0019	7.89E-33	145.2652827
ALM	rs77006977	C	T	0.4525	-0.0287	0.0019	2.42E-52	228.1679615
ALM	rs77083017	G	A	0.1519	-0.0158	0.0026	1.67E-09	36.92883004
ALM	rs77092698	C	T	0.6164	0.0148	0.0019	1.59E-14	60.67563075
ALM	rs7723123	T	C	0.6585	-0.0139	0.002	2.56E-12	48.30228544
ALM	rs7726563	A	C	0.4345	-0.0275	0.0019	2.57E-47	209.4866041
ALM	rs77472398	G	A	0.6879	-0.0192	0.0021	6.84E-21	83.59146542
ALM	rs77569039	A	G	0.5145	-0.0149	0.0019	2.82E-15	61.49834178
ALM	rs77588874	G	A	0.6995	0.0318	0.0021	5.27E-54	229.3051039
ALM	rs7764584	T	C	0.5164	0.0179	0.0019	2.28E-21	88.75583843
ALM	rs77695349	T	C	0.8012	-0.0152	0.0024	1.36E-10	40.11093294
ALM	rs7769535	T	C	0.3854	-0.0212	0.0019	6.94E-28	124.4980619
ALM	rs77698183	T	C	0.3417	-0.0184	0.002	4.83E-20	84.63962403
ALM	rs77704739	T	C	0.5371	-0.0141	0.0019	1.01E-13	55.07177753

ALM	rs77749060	T	C	0.3836	-0.0124	0.0019	1.22E-10	42.59260858
ALM	rs77806914	C	T	0.0412	-0.0323	0.0048	1.6E-11	45.28148288
ALM	rs77830963	C	T	0.1329	-0.0178	0.0028	2.25E-10	40.41308579
ALM	rs7788236	T	C	0.3454	-0.0145	0.002	3.85E-13	52.56226651
ALM	rs77994307	C	T	0.6495	0.0473	0.002	8.18E-126	559.3200155
ALM	rs78175593	C	T	0.136	0.0246	0.0028	3.67E-19	77.18843263
ALM	rs78178106	A	G	0.6064	-0.0165	0.0019	1.03E-17	75.41517747
ALM	rs78183690	G	C	0.0245	0.0347	0.0061	1.36E-08	32.35916827
ALM	rs78375732	T	G	0.3114	-0.0131	0.002	1.35E-10	42.90230943
ALM	rs78413600	C	G	0.8966	-0.0345	0.0032	7.51E-28	116.2348352
ALM	rs78430069	G	A	0.4275	-0.011	0.0019	7.9E-09	33.51785665
ALM	rs7847036	C	A	0.0906	-0.0328	0.0033	2.36E-23	98.79111305
ALM	rs78562459	C	A	0.3953	-0.011	0.0019	1.09E-08	33.51785665
ALM	rs78688524	G	A	0.0945	-0.0269	0.0032	6.2E-17	70.66472517
ALM	rs78772064	C	T	0.5509	-0.0123	0.0019	7.53E-11	41.9084011
ALM	rs78888579	G	A	0.5195	0.0152	0.0019	4.94E-16	63.99971571
ALM	rs78943924	C	T	0.5051	0.0148	0.0019	7.69E-15	60.67563075
ALM	rs78965803	G	A	0.387	0.0228	0.0019	4.65E-32	143.9993603
ALM	rs79010437	T	C	0.341	0.0124	0.002	4.78E-10	38.43982925
ALM	rs79030537	T	C	0.3266	-0.0132	0.002	9.69E-11	43.5598065
ALM	rs7904721	T	C	0.6167	-0.0158	0.0019	3.98E-16	69.15204739
ALM	rs79113308	A	G	0.2392	-0.0293	0.0022	7.16E-39	177.373179
ALM	rs79139572	G	A	0.6944	0.0243	0.0021	4.9E-32	133.8973644
ALM	rs79147753	T	G	0.3673	-0.0145	0.002	1.39E-13	52.56226651
ALM	rs79149870	C	G	0.2298	-0.0144	0.0023	2.14E-10	39.19831359
ALM	rs79369066	T	C	0.4812	0.0302	0.0019	3.44E-57	252.641537
ALM	rs7938808	T	C	0.682	0.0196	0.002	6.93E-22	96.03957339
ALM	rs79425445	A	G	0.0627	0.0301	0.004	2.88E-14	56.62537347
ALM	rs7943376	C	G	0.2527	0.0195	0.0022	2.85E-19	78.5637006
ALM	rs79455560	G	A	0.0603	-0.0318	0.004	8.46E-16	63.20221925
ALM	rs79461529	G	A	0.2311	-0.0303	0.0022	8.3E-42	189.6871739
ALM	rs79484360	C	T	0.0873	-0.0369	0.0033	2.87E-28	125.0325024
ALM	rs79485136	C	T	0.3274	-0.0435	0.002	2.09E-103	473.0603986
ALM	rs79580290	G	A	0.314	-0.0214	0.002	6.04E-26	114.4894914
ALM	rs79605592	C	G	0.1082	-0.0203	0.0031	2.87E-11	42.88118309
ALM	rs79614381	G	A	0.5913	0.0108	0.0019	1.55E-08	32.31010578
ALM	rs79673533	G	A	0.7765	0.0136	0.0024	1.82E-08	32.11096847
ALM	rs79674682	T	C	0.1388	0.0167	0.0027	1.15E-09	38.25634584
ALM	rs79695844	G	A	0.5242	0.0192	0.0019	1.95E-24	102.1158899
ALM	rs79754947	C	A	0.0441	0.0318	0.0046	4.09E-12	47.78995785
ALM	rs7983171	A	G	0.2951	-0.0133	0.0021	1.67E-10	40.11093294
ALM	rs79963825	C	T	0.6059	0.0111	0.0019	9.32E-09	34.1300423
ALM	rs80025215	C	G	0.0102	0.0672	0.0094	9.92E-13	51.10706134
ALM	rs80031568	C	T	0.5847	0.0163	0.0019	2.11E-17	73.59801102
ALM	rs8004632	G	A	0.7779	-0.0182	0.0023	2.05E-15	62.61597894
ALM	rs80075573	T	A	0.3844	0.0236	0.0019	3.45E-34	154.2818631
ALM	rs80182606	T	A	0.1649	0.0163	0.0026	1.97E-10	39.30307985
ALM	rs80248317	T	C	0.6749	-0.0129	0.002	1.12E-10	41.6023152
ALM	rs8089092	G	T	0.3192	0.0166	0.0021	7.33E-16	62.48498321

ALM	rs8101032	C	T	0.5799	0.0118	0.002	3.51E-09	34.80984537
ALM	rs8122151	G	C	0.1218	0.0198	0.0029	7.71E-12	46.61572634
ALM	rs8124843	C	G	0.239	0.0291	0.0022	3.92E-39	174.9599666
ALM	rs8142764	C	G	0.2976	-0.0123	0.0021	3.59E-09	34.30597006
ALM	rs8192388	G	C	0.2513	0.0188	0.0022	9.17E-18	73.02446901
ALM	rs838427	G	A	0.4002	-0.0151	0.0019	3.51E-15	63.16038426
ALM	rs871772	G	A	0.2643	0.0276	0.0022	2.49E-37	157.3877306
ALM	rs884186	A	T	0.6994	-0.0215	0.0021	1.87E-25	104.8181285
ALM	rs9304105	T	G	0.4565	0.0111	0.0019	7.68E-09	34.1300423
ALM	rs939521	C	A	0.1516	0.018	0.0026	1.13E-11	47.92878118
ALM	rs9472191	A	G	0.4738	0.0149	0.0019	9.31E-15	61.49834178
ALM	rs9556323	G	A	0.858	-0.0156	0.0027	1.16E-08	33.38256776
ALM	rs9556581	T	G	0.2303	0.0158	0.0023	3.37E-12	47.19071665
ALM	rs9590329	A	G	0.2922	0.012	0.0021	8.66E-09	32.65291618
ALM	rs9619072	C	T	0.8029	0.0211	0.0024	2.52E-18	77.29305944
ALM	rs9659043	C	G	0.8502	0.0183	0.0026	4.19E-12	49.53972077
ALM	rs9714631	T	C	0.1095	0.0168	0.003	2.64E-08	31.3598607
ALM	rs9857976	G	A	0.4303	0.0114	0.0019	2.36E-09	35.99984009
Walking speed	rs10750025	C	T	0.681757	-0.00836	0.001365	9.4E-10	37.5098531
Walking speed	rs10828258	A	G	0.319319	-0.00933	0.001359	6.7E-12	47.1327181
Walking speed	rs10862220	T	G	0.674897	0.008408	0.001351	4.8E-10	38.7322583
Walking speed	rs10883618	G	A	0.372248	0.00781	0.001308	2.4E-09	35.6520651
Walking speed	rs11077815	T	C	0.623657	-0.00713	0.001307	0.000000048	29.7595251
Walking speed	rs11152989	C	T	0.312264	-0.00751	0.001367	0.000000039	30.1815095
Walking speed	rs11548200	T	C	0.066034	-0.01604	0.002552	3.3E-10	39.5044027
Walking speed	rs11682482	T	G	0.680801	0.008019	0.001356	3.3E-09	34.9718945
Walking speed	rs11732213	T	C	0.195605	0.009161	0.001596	9.4E-09	32.947169
Walking speed	rs11761141	T	G	0.325697	-0.0079	0.001351	0.000000005	34.1933456
Walking speed	rs11848096	T	C	0.386669	-0.00751	0.001307	9.2E-09	33.016179
Walking speed	rs12042959	A	G	0.14387	0.012566	0.001813	4.1E-12	48.0392937
Walking speed	rs12461902	G	A	0.329608	-0.00801	0.001357	3.6E-09	34.8420298
Walking speed	rs12747822	T	A	0.09792	0.011768	0.00214	0.000000038	30.239589
Walking speed	rs12883788	C	T	0.459747	-0.0078	0.001274	9.4E-10	37.4842185
Walking speed	rs144333966	A	G	0.014646	0.030461	0.005446	0.000000022	31.2846392
Walking speed	rs1592	A	C	0.395455	0.007451	0.001288	7.3E-09	33.4653608
Walking speed	rs2037735	C	T	0.120628	-0.01127	0.001943	6.7E-09	33.6434359
Walking speed	rs205262	A	G	0.26893	-0.00871	0.001427	0.000000001	37.2551636
Walking speed	rs2170670	G	A	0.606925	-0.00709	0.001297	0.000000047	29.8820666
Walking speed	rs2297600	T	G	0.17194	-0.01156	0.001682	6.2E-12	47.2347691
Walking speed	rs2439823	A	G	0.545578	-0.00728	0.001275	0.000000011	32.601719
Walking speed	rs2602731	A	G	0.681058	-0.00767	0.001369	0.000000021	31.3893226
Walking speed	rs2644135	C	G	0.655936	0.007512	0.001332	0.000000017	31.805397
Walking speed	rs2645979	G	A	0.357204	0.008618	0.00132	6.7E-11	42.6248858
Walking speed	rs273512	C	T	0.405352	-0.00967	0.001291	6.9E-14	56.1046135
Walking speed	rs28519617	T	G	0.269813	-0.00813	0.001434	0.000000014	32.1425923
Walking speed	rs35711462	A	G	0.511213	-0.00717	0.001269	0.000000017	31.9236969
Walking speed	rs4109292	G	A	0.493943	0.007353	0.001268	6.6E-09	33.6270474
Walking speed	rs4516268	C	A	0.193704	0.00983	0.001607	9.5E-10	37.4173816
Walking speed	rs45583845	C	G	0.034279	-0.0199	0.003633	0.000000043	30.003607

Walking speed	rs4643373	T	C	0.299973	0.007727	0.001382	0.000000023	31.2610708
Walking speed	rs4715208	A	G	0.752863	-0.00838	0.001466	0.000000011	32.6751965
Walking speed	rs4839898	G	A	0.109786	0.013056	0.00206	2.3E-10	40.1683464
Walking speed	rs55680124	C	T	0.154913	-0.01074	0.00175	8.3E-10	37.6643587
Walking speed	rs57800857	A	C	0.36486	0.008676	0.001322	5.3E-11	43.0699421
Walking speed	rs6763292	A	G	0.780939	0.009588	0.001531	3.8E-10	39.2196561
Walking speed	rs7789719	T	C	0.782126	0.008542	0.001537	0.000000027	30.8865578
Walking speed	rs7795394	T	A	0.624965	0.009305	0.001309	1.2E-12	50.5302647
Walking speed	rs8010773	T	C	0.38208	-0.00817	0.001301	3.4E-10	39.4355254
Walking speed	rs8011870	G	A	0.288257	-0.00783	0.001406	0.000000026	31.0135152
Walking speed	rs819167	A	G	0.936466	-0.0158	0.002593	1.1E-09	37.128487
Walking speed	rs830627	G	A	0.417471	0.007421	0.001285	7.7E-09	33.3516035
Walking speed	rs9366651	G	T	0.507037	0.009485	0.001271	8.6E-14	55.6905723
Walking speed	rs9783304	G	T	0.689164	0.007636	0.001368	0.000000024	31.1571522

EA, effect allele; EAF, effect allele frequency; NEA, non-effect allele; SE, standard error; ALM, Appendicular lean mass.

Supplementary Table 10. Genetic association between sarcopenia-related traits and falls

Exposure	method	nsnp	b	se	or	or_lci95	or_uci95	pval
Hand strength	Inverse variance weighted	125	-0.06763736	0.0556369	0.93459934	0.83804316	1.04228035	0.22410208
	MR PRESSO	125	-0.06763736	0.05591461	0.9345993	0.8375871	1.042848	0.22641149
	MR Egger	125	-0.14704078	0.21850638	0.86325877	0.56252861	1.32476056	0.50225046
	Weighted median	125	-0.03893091	0.0755898	0.96181716	0.82937165	1.1154134	0.60653301
	Weighted mode	125	-0.04841282	0.17263038	0.95274039	0.67924841	1.33635096	0.77960571
Appendicular lean mass	Inverse variance weighted	616	-0.03629453	0.01920935	0.96435622	0.92872291	1.00135672	0.05883557
	MR PRESSO	616	-0.03629453	0.019244997	0.9643562	0.928658	1.001427	0.0593057
	MR Egger	616	-0.06072603	0.04233136	0.94108103	0.86615145	1.02249267	0.15192736
	Weighted median	616	-0.05126071	0.03180058	0.95003095	0.89262398	1.01112991	0.10697446
	Weighted mode	616	-0.02586158	0.0358764	0.97446997	0.90830111	1.04545916	0.47127448
Walking speed	Inverse variance weighted	45	-0.450732042	0.139379548	0.637161552	0.484849883	0.8373207	0.001221351
	MR PRESSO	45	-0.450732042	0.147973906	0.6371616	0.476751	0.8515448	0.002318895
	MR Egger	45	-1.334359687	0.567745305	0.263326733	0.086540697	0.801252709	0.023417319
	Weighted median	45	-0.583305054	0.159031936	0.558050927	0.408604474	0.762157189	0.0002446
	Weighted mode	45	-0.72141482	0.347673913	0.486064076	0.245893764	0.960814467	0.043868167

Supplementary Table 11. Testing for heterogeneity and pleiotropy

Exposure	egger_intercept	se_intercept	pval_intercept	Q_IVW	Q_df_IVW	Q_pval_IVW
Hand strength	0.000957254	0.00254678	0.707662387	162.9826778	124	0.010823735
Appendicular lean mass	0.000646736	0.000998431	0.51738701	743.2767044	615	0.000281276
Walking speed	0.008125747	0.005066747	0.116092297	76.03347992	44	0.001926025

Supplementary Information: Formulas for converting RR or HR values into ORs

All non-odds-ratio point estimates were converted to ORs (the most frequently reported type) using one of the following formulas:

RR to OR

$$\mathrm{OR} = \frac{\mathrm{RR} * (1 - \mathrm{K})}{1 - \mathrm{RR} * \mathrm{K}}$$

HR to OR

$$\mathrm{OR} = \frac{\left(1 - \mathrm{e}^{\mathrm{HR}*\ln(1-\mathrm{K})}\right) * (1 - \mathrm{K})}{\mathrm{K} * \mathrm{e}^{\mathrm{HR}*\ln(1-\mathrm{K})}}$$

OR: odds ratio; RR: relative risk; HR: hazard ratio; K: Background risk, represents the probability of an event occurring in the non-exposed group.

Appendix

Supplementary Figure 1. Forest plots of meta-analysis odds ratio for falls in categorical sarcopenia-related traits in cohort study (A) Hand strength; (B) Appendicular lean mass; (C) Walking speed.

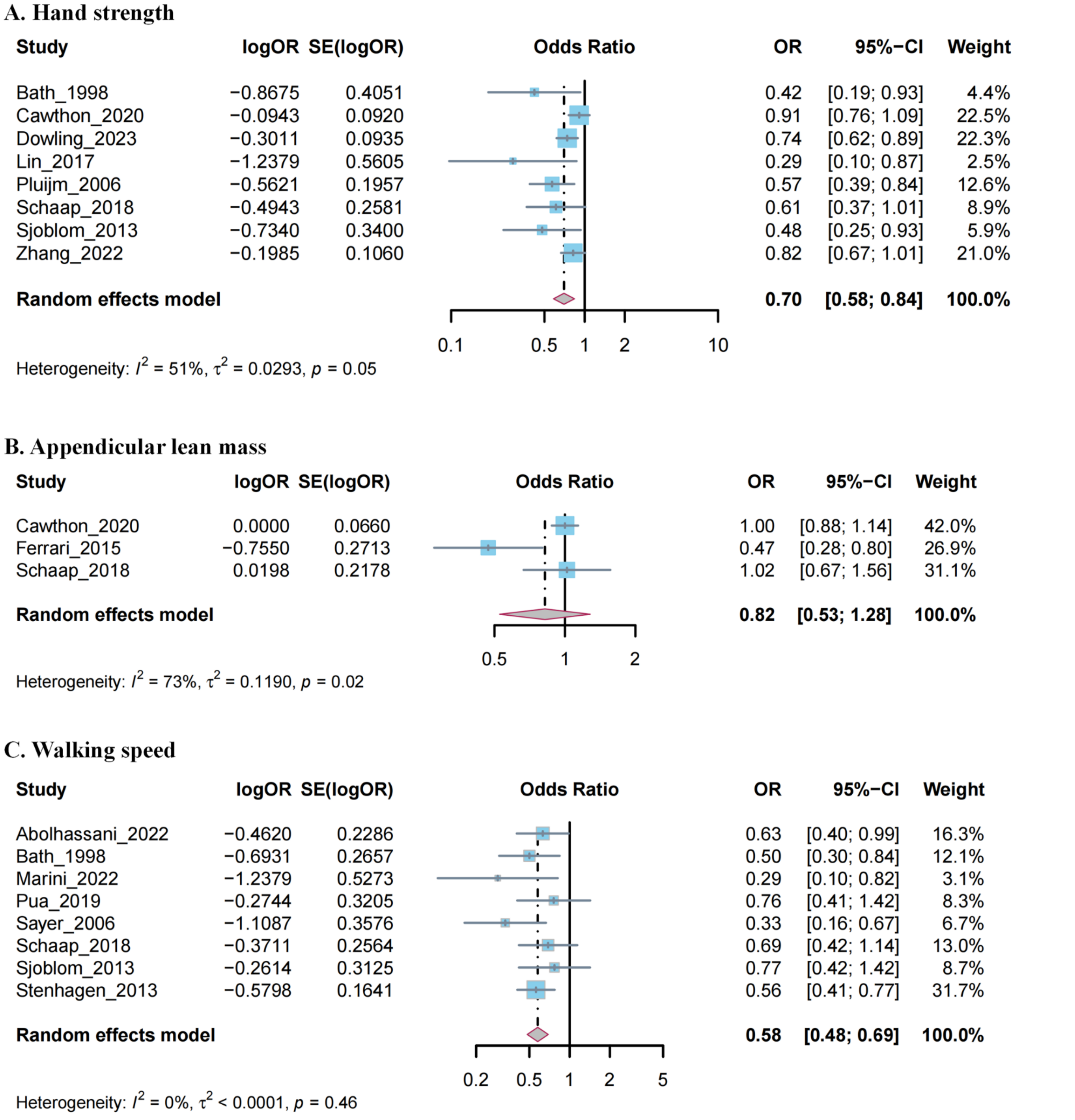

Forest plot showing the individual and summary random effects estimates for associations between higher sarcopenia-related traits group and falls compared to the lower group in cohort study. The blue squares represent the weight of each study, and the gray bars show the 95% CIs. The gray and maroon diamond shows the pooled odds ratio.

Supplementary Figure 2. Forest plots of subgroup meta-analysis odds ratio for falls in continuous sarcopenia-related traits in cohort study.

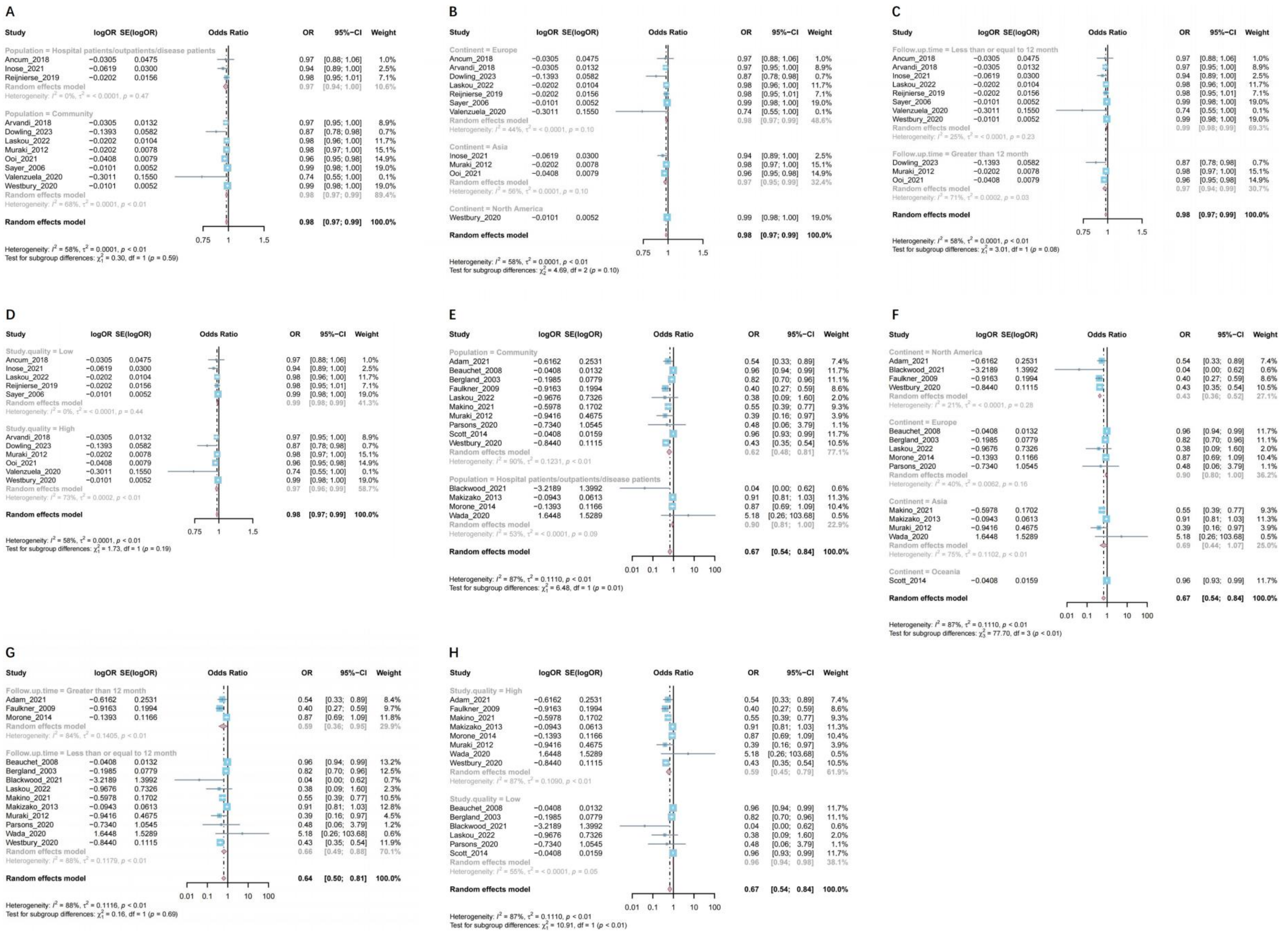

Forest plot showing the subgroup meta-analysis for associations between continuous sarcopenia-related traits and falls in cohort study. The blue squares represent the weight of each study, and the gray bars show the 95% CIs. The gray and maroon diamond shows the pooled odds ratio. Hand strength is stratified by (A) population; (B) continent; (C) Follow-up time; (D) Study quality and walking speed stratified by (E) population; (F) continent; (G) Follow-up time; (H) Study quality.

Supplementary Figure 3. Forest plots of subgroup meta-analysis odds ratio for falls in categorical sarcopenia-related traits in cohort study.

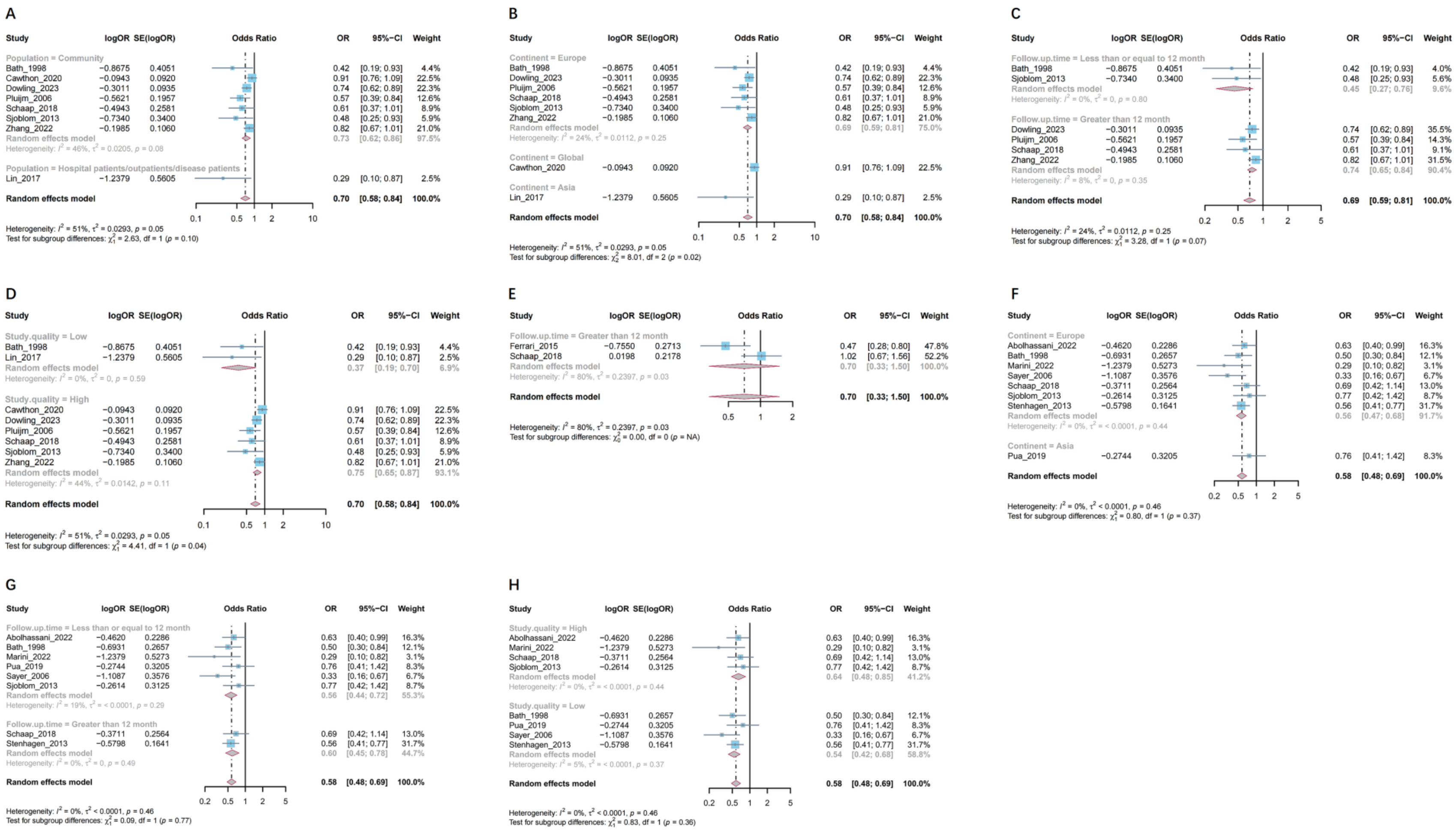

Forest plot showing the subgroup meta-analysis for associations between categorical sarcopenia-related traits and falls in cohort study. The blue squares represent the weight of each study, and the gray bars show the 95% CIs. The gray and maroon diamond shows the pooled odds ratio. Hand strength is stratified by (A) population; (B) continent; (C) Follow-up time; (D) Study quality, appendicular lean mass stratified by (E) Follow-up time and walking speed stratified by (F) continent; (G) Follow-up time; (H) Study quality.

Supplementary Figure 4. Forest plots of sex subgroup meta-analysis odds ratio for falls in sarcopenia-related traits in cohort study.

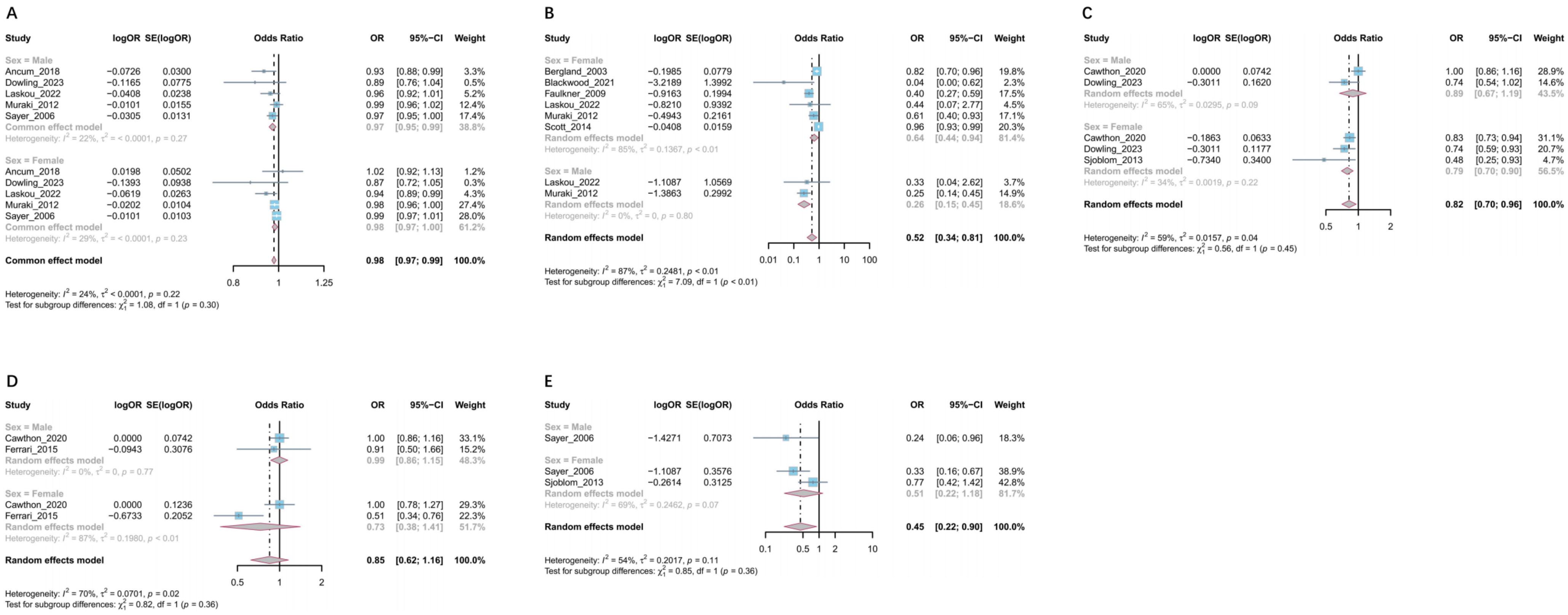

Forest plot showing the sex subgroup meta-analysis for associations between sarcopenia-related traits and falls in cohort study. The blue squares represent the weight of each study, and the gray bars show the 95% CIs. The gray and maroon diamond shows the pooled odds ratio. Continuous sarcopenia-related traits is consisted of (A) Hand strength; (B) Walking speed, and categorical sarcopenia-related traits is consisted of (C) Hand strength; (D) Appendicular lean mass; (E) Walking speed.

Supplementary Figure 5. Forest plots of walking test distance subgroup meta-analysis odds ratio for falls in sarcopenia-related traits in cohort study.

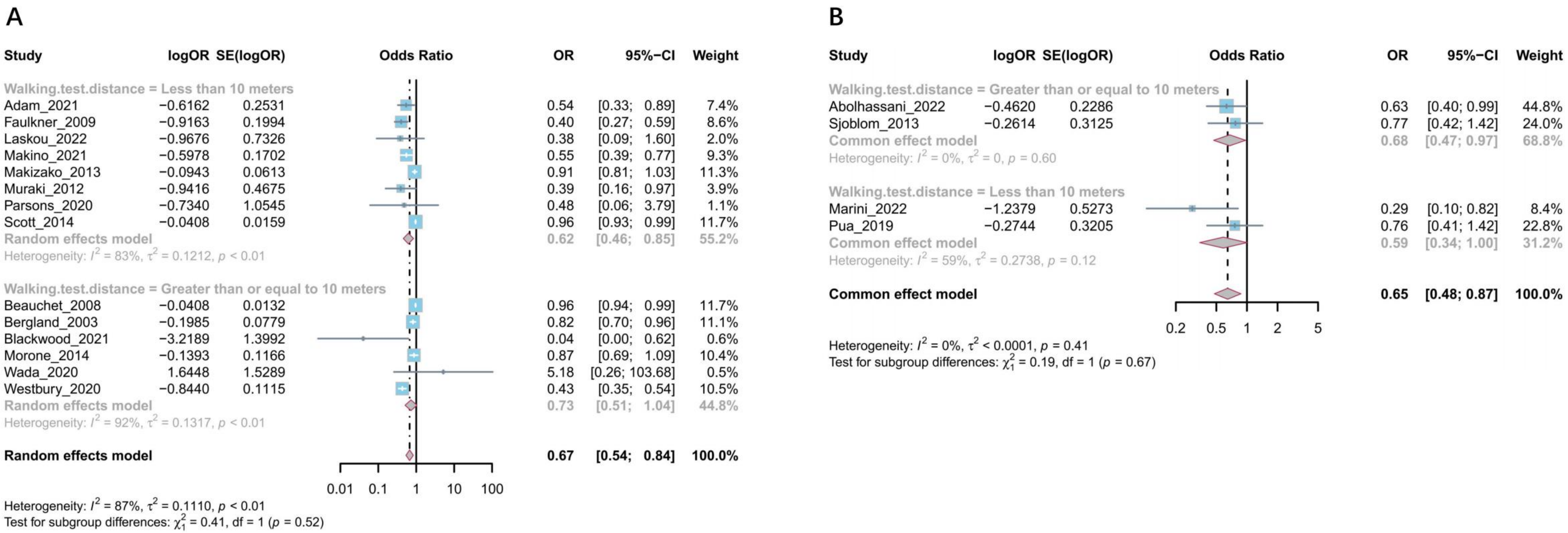

Forest plot showing the walking test distance subgroup meta-analysis for associations between walking speed and falls in cohort study. The blue squares represent the weight of each study, and the gray bars show the 95% CIs. The gray and maroon diamond shows the pooled odds ratio. (A) Continuous walking speed; (B) Categorical walking speed.

Supplementary Figure 6. Forest plots of omitting sensitivity meta-analysis odds ratio for falls in sarcopenia-related traits in cohort study.

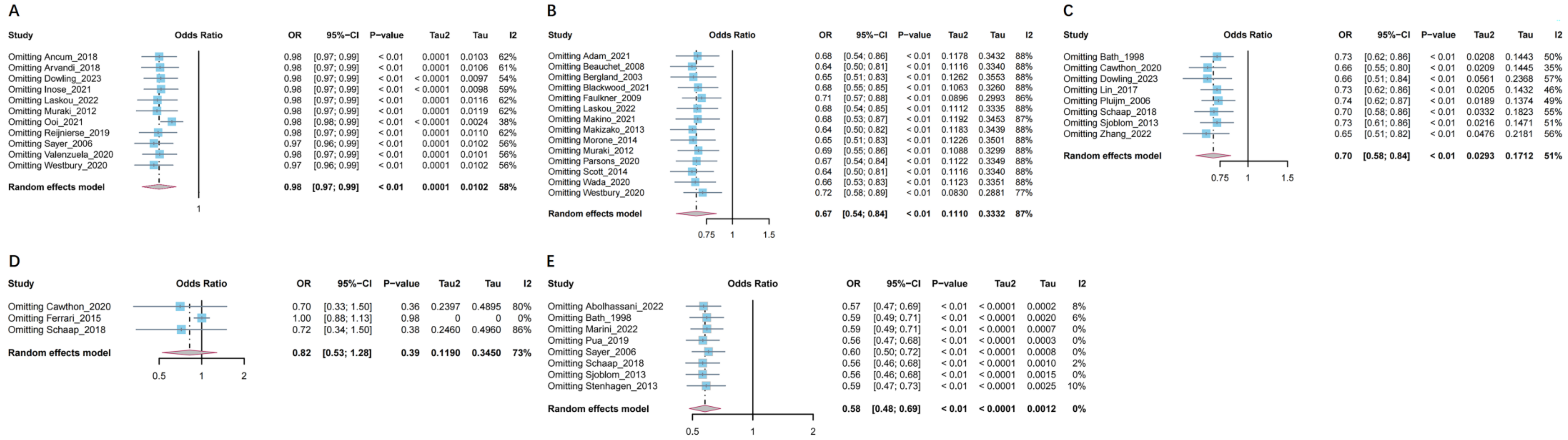

Forest plot showing the omitting sensitivity meta-analysis for associations between sarcopenia-related traits and falls in cohort study. The point estimation represents the sum of the effects of the remaining studies after excluding that study, and the gray bars show the 95% CIs. The gray and maroon diamond shows the pooled odds ratio. Continuous sarcopenia-related traits is consisted of (A) Hand strength; (B) Walking speed, and categorical sarcopenia-related traits is consisted of (C) Hand strength; (D) Appendicular lean mass; (E) Walking speed.

Supplementary Figure 7. Funnel plots for falls in sarcopenia-related traits in meta-analysis.

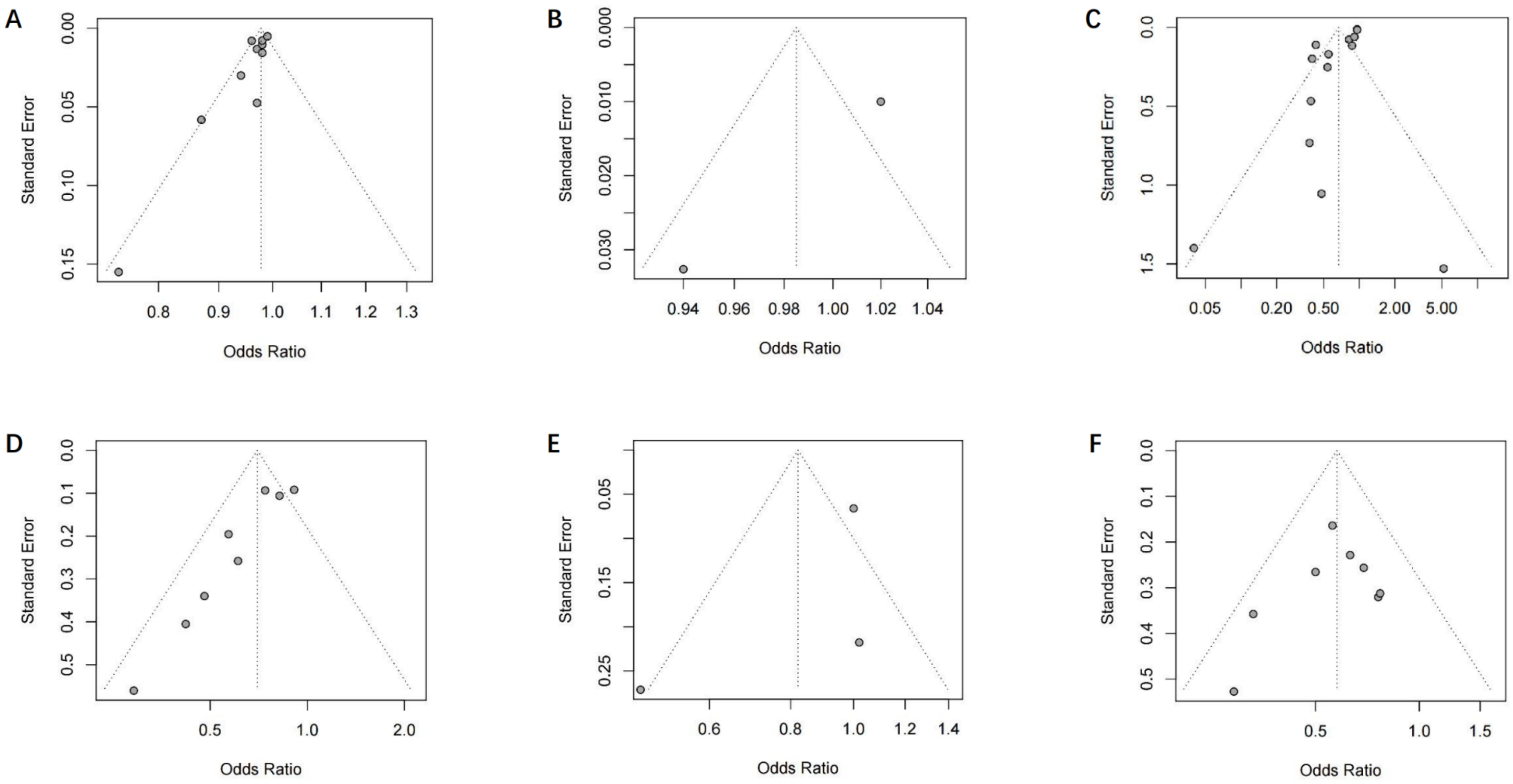

Risk of publication bias analysis for falls in sarcopenia-related traits, continuous sarcopenia-related traits is consisted of (A) Hand strength; (B) Appendicular lean mass; (C) Walking speed, and categorical sarcopenia-related traits is consisted of (D) Hand strength; (E) Appendicular lean mass; (F) Walking speed.

Supplementary Figure 8. Directed acyclic graph (DAG) of sarcopenia-related traits and falls.

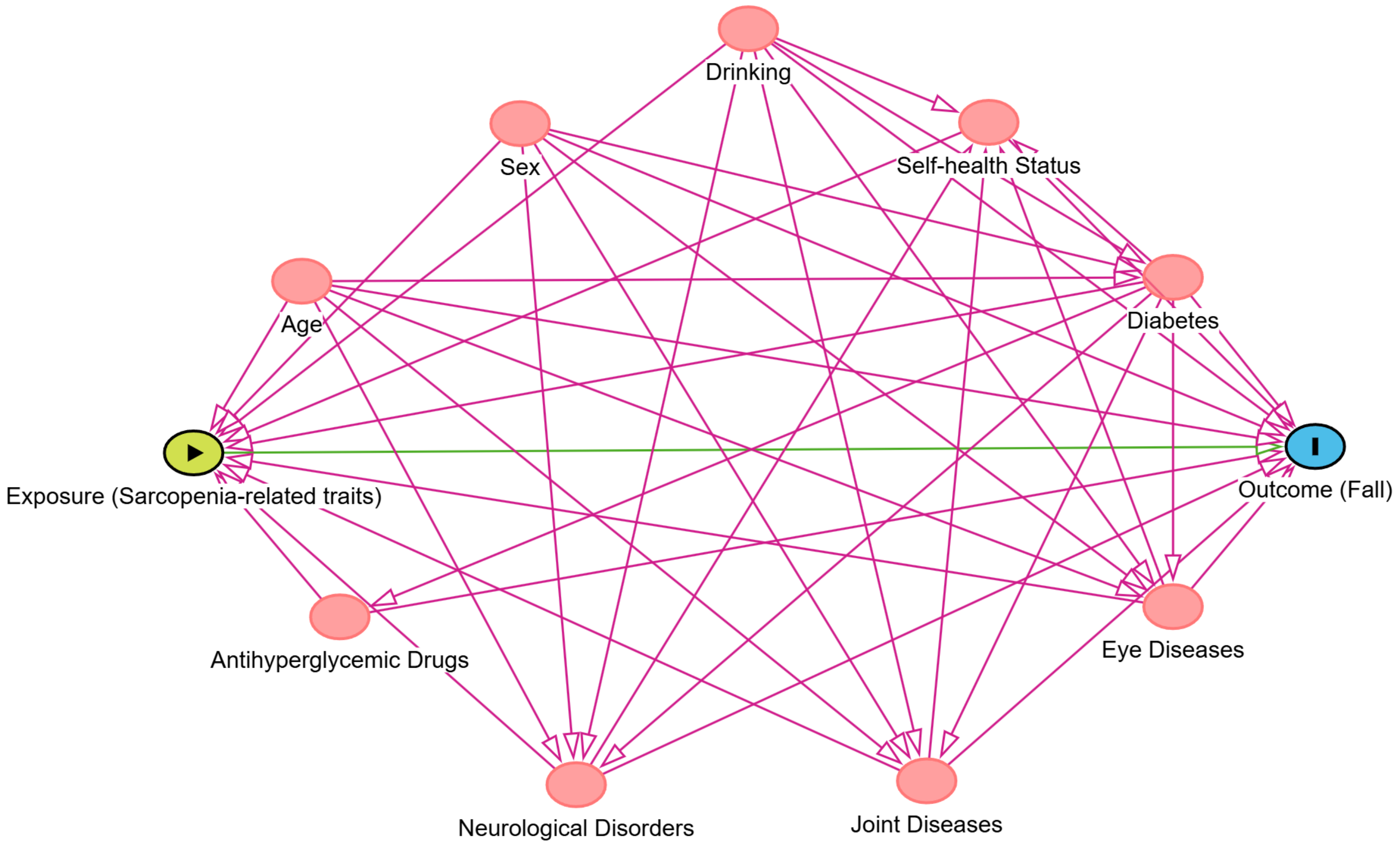

In the MR analysis, we retained SNPs associated with age and sex because these SNPs are genetically randomly assigned and represent biologically immutable baseline characteristics that are critical for accurate causal inference.

Supplementary Figure 9. Scatter plots for falls in sarcopenia-related traits in MR analysis.

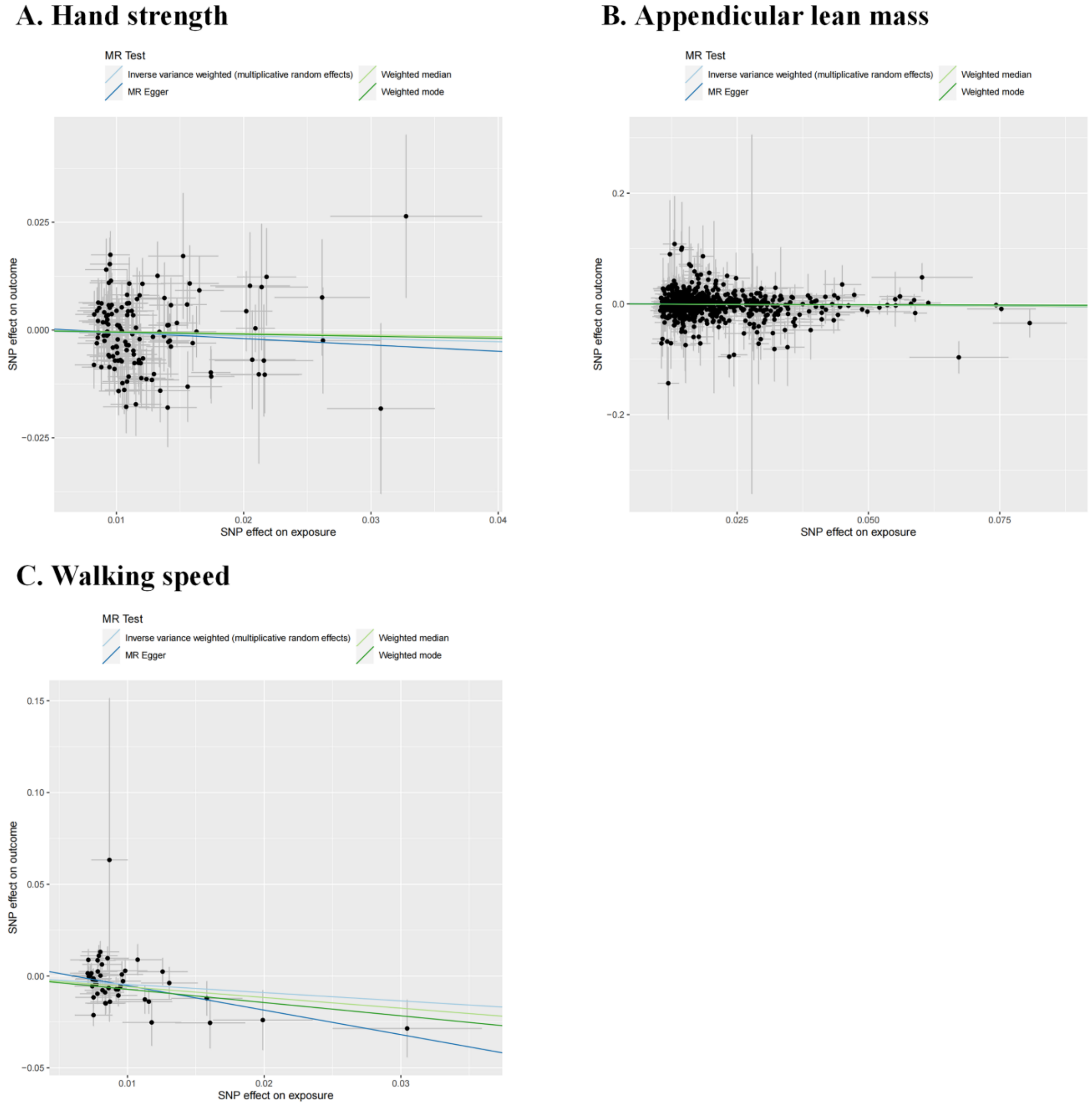

The scatter plots for falls in sarcopenia-related traits is consisted of (A) Hand strength; (B) Appendicular lean mass; (C) Walking speed.

Supplementary Figure 10. Probability curves for falls in sarcopenia-related traits.

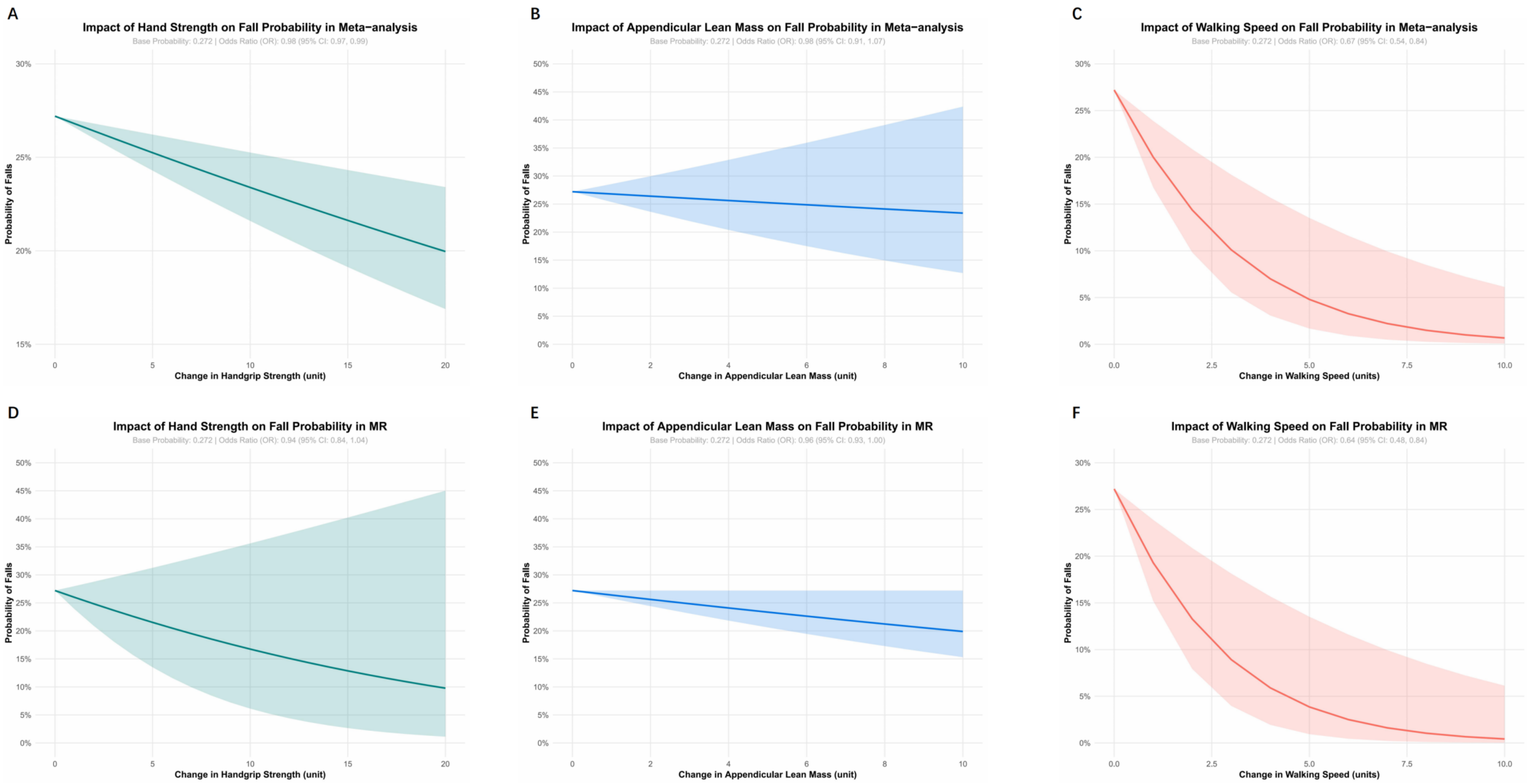

The probability curves for falls in sarcopenia-related traits, meta-analysis is consisted of (A) Hand strength; (B) Appendicular lean mass; (C)Walking speed, and MR is consisted of (D) Hand strength; (E) Appendicular lean mass; (F)Walking speed.

PRISMA 2020 Checklist

Section and Topic	Item #	Checklist item	Location where item is reported
**TITLE**			
Title	1	Identify the report as a systematic review.	N/A
**ABSTRACT**			
Abstract	2	See the PRISMA 2020 for Abstracts checklist.	1
**INTRODUCTION**			
Rationale	3	Describe the rationale for the review in the context of existing knowledge.	1-2
Objectives	4	Provide an explicit statement of the objective(s) or question(s) the review addresses.	1-2
**METHODS**			
Eligibility criteria	5	Specify the inclusion and exclusion criteria for the review and how studies were grouped for the syntheses.	2
Information sources	6	Specify all databases, registers, websites, organisations, reference lists and other sources searched or consulted to identify studies. Specify the date when each source was last searched or consulted.	2
Search strategy	7	Present the full search strategies for all databases, registers and websites, including any filters and limits used.	2
Selection process	8	Specify the methods used to decide whether a study met the inclusion criteria of the review, including how many reviewers screened each record and each report retrieved, whether they worked independently, and if applicable, details of automation tools used in the process.	2
Data collection process	9	Specify the methods used to collect data from reports, including how many reviewers collected data from each report, whether they worked independently, any processes for obtaining or confirming data from study investigators, and if applicable, details of automation tools used in the process.	2
Data items	10a	List and define all outcomes for which data were sought. Specify whether all results that were compatible with each outcome domain in each study were sought (e.g. for all measures, time points, analyses), and if not, the methods used to decide which results to collect.	2
	10b	List and define all other variables for which data were sought (e.g. participant and intervention characteristics, funding sources). Describe any assumptions made about any missing or unclear information.	2
Study risk of bias assessment	11	Specify the methods used to assess risk of bias in the included studies, including details of the tool(s) used, how many reviewers assessed each study and whether they worked independently, and if applicable, details of automation tools used in the process.	3
Effect measures	12	Specify for each outcome the effect measure(s) (e.g. risk ratio, mean difference) used in the synthesis or presentation of results.	3
Synthesis methods	13a	Describe the processes used to decide which studies were eligible for each synthesis (e.g. tabulating the study intervention characteristics and comparing against the planned groups for each synthesis (item #5)).	N/A
	13b	Describe any methods required to prepare the data for presentation or synthesis, such as handling of missing summary statistics, or data conversions.	3
	13c	Describe any methods used to tabulate or visually display results of individual studies and syntheses.	3
	13d	Describe any methods used to synthesize results and provide a rationale for the choice(s). If meta-analysis was performed, describe the model(s), method(s) to identify the presence and extent of statistical heterogeneity, and software package(s) used.	3
	13e	Describe any methods used to explore possible causes of heterogeneity among study results (e.g. subgroup analysis, meta-regression).	3
	13f	Describe any sensitivity analyses conducted to assess robustness of the synthesized results.	3
Reporting bias assessment	14	Describe any methods used to assess risk of bias due to missing results in a synthesis (arising from reporting biases).	3
Certainty assessment	15	Describe any methods used to assess certainty (or confidence) in the body of evidence for an outcome.	N/A

PRISMA 2020 Checklist

Section and Topic	Item #	Checklist item	Location where item is reported
**RESULTS**			
Study selection	16a	Describe the results of the search and selection process, from the number of records identified in the search to the number of studies included in the review, ideally using a flow diagram.	Figure 2
	16b	Cite studies that might appear to meet the inclusion criteria, but which were excluded, and explain why they were excluded.	N/A
Study characteristics	17	Cite each included study and present its characteristics.	4, Table 1
Risk of bias in studies	18	Present assessments of risk of bias for each included study.	4
Results of individual studies	19	For all outcomes, present, for each study: (a) summary statistics for each group (where appropriate) and (b) an effect estimate and its precision (e.g. confidence/credible interval), ideally using structured tables or plots.	Figure 3, Appendix
Results of syntheses	20a	For each synthesis, briefly summarise the characteristics and risk of bias among contributing studies.	4-5, Appendix
	20b	Present results of all statistical syntheses conducted. If meta-analysis was done, present for each the summary estimate and its precision (e.g. confidence/credible interval) and measures of statistical heterogeneity. If comparing groups, describe the direction of the effect.	4-6
	20c	Present results of all investigations of possible causes of heterogeneity among study results.	6-7
	20d	Present results of all sensitivity analyses conducted to assess the robustness of the synthesized results.	6-7
Reporting biases	21	Present assessments of risk of bias due to missing results (arising from reporting biases) for each synthesis assessed.	7
Certainty of evidence	22	Present assessments of certainty (or confidence) in the body of evidence for each outcome assessed.	N/A
**DISCUSSION**			
Discussion	23a	Provide a general interpretation of the results in the context of other evidence.	8-9
	23b	Discuss any limitations of the evidence included in the review.	10
	23c	Discuss any limitations of the review processes used.	10
	23d	Discuss implications of the results for practice, policy, and future research.	10
**OTHER INFORMATION**			
Registration and protocol	24a	Provide registration information for the review, including register name and registration number, or state that the review was not registered.	2
	24b	Indicate where the review protocol can be accessed, or state that a protocol was not prepared.	2
	24c	Describe and explain any amendments to information provided at registration or in the protocol.	2
Support	25	Describe sources of financial or non-financial support for the review, and the role of the funders or sponsors in the review.	10-11
Competing interests	26	Declare any competing interests of review authors.	11
Availability of data, code and other materials	27	Report which of the following are publicly available and where they can be found: template data collection forms; data extracted from included studies; data used for all analyses; analytic code; any other materials used in the review.	11

From: Page MJ, McKenzie JE, Bossuyt PM, Boutron I, Hoffmann TC, Mulrow CD, et al. The PRISMA 2020 statement: an updated guideline for reporting systematic reviews. BMJ 2021;372:n71. doi: 10.1136/bmj.n71

STROBE-MR checklist of recommended items to address in reports of Mendelian randomization studies[1 2]

Item No.	Section		Checklist item	Page No.	Relevant text from manuscript
1	**TITLE and ABSTRACT**		Indicate Mendelian randomization (MR) as the study's design in the title and/or the abstract if that is a main purpose of the study	1	Title, Abstract
	**INTRODUCTION**				
2	**Background**		Explain the scientific background and rationale for the reported study. What is the exposure? Is a potential causal relationship between exposure and outcome plausible? Justify why MR is a helpful method to address the study question	1-2	Background: paragraphs 1-3
3	**Objectives**		State specific objectives clearly, including pre-specified causal hypotheses (if any). State that MR is a method that, under specific assumptions, intends to estimate causal effects	2	Background: paragraphs 3-4
	**METHODS**				
4	**Study design and data sources**		Present key elements of the study design early in the article. Consider including a table listing sources of data for all phases of the study. For each data source contributing to the analysis, describe the following:		
		a)	Setting: Describe the study design and the underlying population, if possible. Describe the setting, locations, and relevant dates, including periods of recruitment, exposure, follow-up, and data collection, when available.	N/A	N/A
		b)	Participants: Give the eligibility criteria, and the sources and methods of selection of participants. Report the sample size, and whether any power or sample size calculations were carried out prior to the main analysis	3	Methods: Mendelian randomization - Data sources, Supplementary Table 8
		c)	Describe measurement, quality control and selection of genetic variants	3-4	Methods: Mendelian randomization - Selection of instrumental variables
		d)	For each exposure, outcome, and other relevant variables, describe methods of assessment and diagnostic criteria for diseases		N/A
		e)	Provide details of ethics committee approval and participant informed consent, if relevant	3	Methods: Mendelian randomization
5	**Assumptions**		Explicitly state the three core IV assumptions for the main analysis (relevance, independence and exclusion restriction) as well assumptions for any additional or sensitivity analysis	3	Methods: Mendelian randomization, Figure 1

6	**Statistical methods: main analysis**		Describe statistical methods and statistics used		
		a)	Describe how quantitative variables were handled in the analyses (i.e., scale, units, model)	4	Methods: Mendelian randomization - Statistical analysis
		b)	Describe how genetic variants were handled in the analyses and, if applicable, how their weights were selected		
		c)	Describe the MR estimator (e.g. two-stage least squares, Wald ratio) and related statistics. Detail the included covariates and, in case of two-sample MR, whether the same covariate set was used for adjustment in the two samples		
		d)	Explain how missing data were addressed		
		e)	If applicable, indicate how multiple testing was addressed		
7	**Assessment of assumptions**		Describe any methods or prior knowledge used to assess the assumptions or justify their validity		N/A
8	**Sensitivity analyses and additional analyses**		Describe any sensitivity analyses or additional analyses performed (e.g. comparison of effect estimates from different approaches, independent replication, bias analytic techniques, validation of instruments, simulations)	4	Methods: Mendelian randomization - Statistical analysis
9	**Software and pre-registration**				
		a)	Name statistical software and package(s), including version and settings used	4	Methods: Mendelian randomization - Statistical analysis
		b)	State whether the study protocol and details were pre-registered (as well as when and where)		N/A
	**RESULTS**				
10	**Descriptive data**				
		a)	Report the numbers of individuals at each stage of included studies and reasons for exclusion. Consider use of a flow diagram		N/A
		b)	Report summary statistics for phenotypic exposure(s), outcome(s), and other relevant variables (e.g. means, SDs, proportions)		
		c)	If the data sources include meta-analyses of previous studies, provide the assessments of heterogeneity across these studies		

		d)	For two-sample MR:<br>i. Provide justification of the similarity of the genetic variant-exposure associations between the exposure and outcome samples<br>ii. Provide information on the number of individuals who overlap between the exposure and outcome studies	3	Methods: Mendelian randomization - Data sources
11	**Main results**				
		a)	Report the associations between genetic variant and exposure, and between genetic variant and outcome, preferably on an interpretable scale	8	Result: Mendelian randomization - Selection of Instrumental Variables, Supplementary Table 9
		b)	Report MR estimates of the relationship between exposure and outcome, and the measures of uncertainty from the MR analysis, on an interpretable scale, such as odds ratio or relative risk per SD difference	8	Result: Mendelian randomization - MR Analysis, Supplementary Table 10
		c)	If relevant, consider translating estimates of relative risk into absolute risk for a meaningful time period		N/A
		d)	Consider plots to visualize results (e.g. forest plot, scatterplot of associations between genetic variants and outcome versus between genetic variants and exposure)		Figure 4
12	**Assessment of assumptions**				
		a)	Report the assessment of the validity of the assumptions	8	Result: Mendelian randomization - MR Analysis, Supplementary Table 11
		b)	Report any additional statistics (e.g., assessments of heterogeneity across genetic variants, such as I^2, Q statistic or E-value)		
13	**Sensitivity analyses and additional analyses**				
		a)	Report any sensitivity analyses to assess the robustness of the main results to violations of the assumptions	8	Result: Mendelian randomization - MR Analysis, Supplementary Table 10
		b)	Report results from other sensitivity analyses or additional analyses		
		c)	Report any assessment of direction of causal relationship (e.g., bidirectional MR)		
		d)	When relevant, report and compare with estimates from non-MR analyses	8	Discussion: Main findings
		e)	Consider additional plots to visualize results (e.g., leave-one-out analyses)		N/A
	**DISCUSSION**				

14	**Key results**	Summarize key results with reference to study objectives	8	Discussion: Main findings
15	**Limitations**	Discuss limitations of the study, taking into account the validity of the IV assumptions, other sources of potential bias, and imprecision. Discuss both direction and magnitude of any potential bias and any efforts to address them	10	Discussion: Strengthens and limitations
16	**Interpretation**			
	a)	Meaning: Give a cautious overall interpretation of results in the context of their limitations and in comparison with other studies	8-9	Discussion: Comparison with other studies
	b)	Mechanism: Discuss underlying biological mechanisms that could drive a potential causal relationship between the investigated exposure and the outcome, and whether the gene-environment equivalence assumption is reasonable. Use causal language carefully, clarifying that IV estimates may provide causal effects only under certain assumptions	9-10	Discussion: Potential Mechanism
	c)	Clinical relevance: Discuss whether the results have clinical or public policy relevance, and to what extent they inform effect sizes of possible interventions	9	Discussion: Clinical implication
17	**Generalizability**	Discuss the generalizability of the study results (a) to other populations, (b) across other exposure periods/timings, and (c) across other levels of exposure	10	Discussion: Strengthens and limitations
	**OTHER INFORMATION**			
18	**Funding**	Describe sources of funding and the role of funders in the present study and, if applicable, sources of funding for the databases and original study or studies on which the present study is based	10-11	Funding
19	**Data and data sharing**	Provide the data used to perform all analyses or report where and how the data can be accessed, and reference these sources in the article. Provide the statistical code needed to reproduce the results in the article, or report whether the code is publicly accessible and if so, where	11	Data availability
20	**Conflicts of Interest**	All authors should declare all potential conflicts of interest	11	Declarations: Conflict of interest

This checklist is copyrighted by the Equator Network under the Creative Commons Attribution 3.0 Unported (CC BY 3.0) license.

1. Skrivankova VW, Richmond RC, Woolf BAR, Yarmolinsky J, Davies NM, Swanson SA, et al. Strengthening the Reporting of Observational Studies in Epidemiology using Mendelian Randomization (STROBE-MR) Statement. JAMA. 2021;under review.

2. Skrivankova VW, Richmond RC, Woolf BAR, Davies NM, Swanson SA, VanderWeele TJ, et al. Strengthening the Reporting of Observational Studies in Epidemiology using Mendelian Randomisation (STROBE-MR): Explanation and Elaboration. BMJ. 2021;375:n2233.
